# Transcriptome Analysis of the Response to NaCl in *Suaeda maritima* Provides an Insight into Salt Tolerance Mechanisms in Halophytes

**DOI:** 10.1371/journal.pone.0163485

**Published:** 2016-09-28

**Authors:** Sachin Ashruba Gharat, Shaifaly Parmar, Subodh Tambat, Madavan Vasudevan, Birendra Prasad Shaw

**Affiliations:** 1 Environmental Biotechnology Laboratory, Institute of Life Sciences, Bhubaneswar, 751023, Odisha, India; 2 Bionivid Technology Private Limited, 3rd Floor, 4C-209, 4th Cross, Near New Horizon College, Kasturi Nagar, Bangalore, 560043, Karnataka, India; Louisiana State University College of Agriculture, UNITED STATES

## Abstract

Although salt tolerance is a feature representative of halophytes, most studies on this topic in plants have been conducted on glycophytes. Transcriptome profiles are also available for only a limited number of halophytes. Hence, the present study was conducted to understand the molecular basis of salt tolerance through the transcriptome profiling of the halophyte *Suaeda maritima*, which is an emerging plant model for research on salt tolerance. Illumina sequencing revealed 72,588 clustered transcripts, including 27,434 that were annotated using BLASTX. Salt application resulted in the 2-fold or greater upregulation of 647 genes and downregulation of 735 genes. Of these, 391 proteins were homologous to proteins in the COGs (cluster of orthologous groups) database, and the majorities were grouped into the poorly characterized category. Approximately 50% of the genes assigned to MapMan pathways showed homology to *S*. *maritima*. The majority of such genes represented transcription factors. Several genes also contributed to cell wall and carbohydrate metabolism, ion relation, redox responses and G protein, phosphoinositide and hormone signaling. Real-time PCR was used to validate the results of the deep sequencing for the most of the genes. This study demonstrates the expression of protein kinase C, the target of diacylglycerol in phosphoinositide signaling, for the first time in plants. This study further reveals that the biochemical and molecular responses occurring at several levels are associated with salt tolerance in *S*. *maritima*. At the structural level, adaptations to high salinity levels include the remodeling of cell walls and the modification of membrane lipids. At the cellular level, the accumulation of glycinebetaine and the sequestration and exclusion of Na^+^ appear to be important. Moreover, this study also shows that the processes related to salt tolerance might be highly complex, as reflected by the salt-induced enhancement of transcription factor expression, including hormone-responsive factors, and that this process might be initially triggered by G protein and phosphoinositide signaling.

## Introduction

Plants, being sessile organisms, had to diversify greatly during the course of evolution to colonize the highly heterogeneous abiotic conditions found across the entire globe, such as extremes of temperature, high and low irradiance levels, high salinity, and drought [1]. Salinity is the most important of the abiotic stresses from the point of view of agriculture because it severely affects the yield of all crops [2], and approximately 6.0% of the land worldwide, more than 800 million ha, is affected by salt [3]. The effects of salt on crops, as shown by the available data, are in contrast to the need to increase food production at a rate of 1.8% per year to feed the increasing world population, which is projected to be 9.5 billion by the year 2050 [4]. Salinity is also unique among the abiotic stresses in that it has two effector components, ionic effects and dehydration, which lead to multiple effects via osmotic stress, induced water deficits, ion toxicity, ionic imbalance, etc. [5]. Because salt affects plants in multiple ways, salinity tolerance is controlled by many genes involved in different processes, such as ion compartmentalization, extrusion and selectivity, the synthesis of compatible solutes, and reactive oxygen species (ROS) scavenging [6–7]. However, these processes are not universal, and depending upon the metabolic background of the species, the relative importance of each individual biochemical pathway in salt tolerance may vary. The quantitative nature of salt tolerance makes it one of the more complex physiological processes yet to be fully understood.

Despite the complexities involved in the processes related to salt tolerance, halophytes are well adapted to high salinity and can produce biomasses varying from 5.2 to 24.6 t/ha, depending on the species, under full seawater irrigation, which is comparable to the range of yields from conventional forage grasses [8]. However, salt tolerance levels in halophytes also vary greatly, ranging from the low salinity levels of coastal lagoons [9] to salinity tolerance levels as high as 1.3 M NaCl (twice the salinity of seawater), as observed in *Salicornia bigelovii* [10–11]. In contrast, the salt tolerance of glycophytes reaches a maximum of 5 g/L of total dissolved solids (TDSs), which is observed in crop plants such as sugar beets (*Beta vulgaris*) and barley (*Hordeum vulgare*), while crops such as rice, which is harmed by 20 to 50 mM NaCl, are among the least salt-tolerance plants [8,12]. Such a gradient and diversity in salt tolerance among plant species not only suggests the functioning of more than one mechanism [10,12] but also indicates varying degrees of interaction among the mechanisms involved in salt tolerance [8]. The mechanisms underlying salt tolerance may include the accumulation of osmolytes, osmotic adjustments [13], the sequestration and compartmentalization of Na^+^ [14], the extrusion and secretion of Na^+^ [15], ROS scavenging [16], and the synthesis of phytohormones [17]. Because salt tolerance is a quantitative trait characteristic of halophytes, it is unlikely that an understanding to the underlying mechanism is going to be successfully addressed through the breeding of crop plants, although quantitative trait loci (QTLs) for the trait have been identified in rice [18]. The use of breeding techniques in understanding the mechanisms of salt tolerance in halophytes is handicapped by the unavailability of parental contrasts for salt tolerance for the use in breeding. Hence, breeding for salt tolerance has remained restricted to crop plants, which are not halophytes, although some do show significant salt tolerance. However, it is believed that a detailed understanding of the mechanisms underlying salt tolerance in plants is likely to be developed only by extending the investigation to halophytes [19]. It has been proposed that any trait that is universally present in halophytes could represent an example of convergent evolution and represent a trait essential for salt tolerance, and such a trait would be a promising candidate for the eventual transfer from halophytes to glycophytes [8,20].

Although salinity tolerance is accomplished via several organized biological processes operating at the cellular level, as stated above, salt tolerance may be far more complex than is currently thought. This is because studies of the response of various plant species to salt have also revealed significant influences of salinity on several physiological and biochemical processes not directly involved in ion relation or osmotic adjustments, such as the activation of signal perception and transduction [2], increases in photosynthesis and energy metabolism [21], changes in protein biosynthesis, folding and decay [22], enhancements in MAPK (mitogen-activated protein kinase) activities [23] and the activation of antioxidative machinery [16]. In addition, the accumulation of heat-shock proteins (HSPs), jasmonic acid-induced proteins (JAIPs) and late embryogenesis abundant (LEA) proteins has also been reported in plants upon exposure to salinity [24–26]. Moreover, salt tolerance is also associated with the enhanced expression of multiple transcription factor families, such as basic leucine zipper (bZIP) [27] and NAC, an abbreviation for NAM (No apical meristem), ATAF (*Arabidopsis* transcription activation factor) and CUC (Cup-shaped cotyledon) [28], which individually regulate the expression of several genes.

The adaptations of plants to abiotic stresses take several forms and are observable from the molecular to cellular and biochemical to physiological levels [8,10,13,19]. However, any adaptive process is preceded by a response to the changing environment, and one such early-stage adaptive response includes dynamic transcriptome changes, the products of which are responsible for enabling the organism to achieve cellular and organismal homeostasis through the co-ordination of various molecular events. The study of changes in transcriptomes in response to an abiotic stress may allow for the identification of the genes related to the stress. With regard to salinity, several such studies have been carried out, but most have focused only on glycophytes [7,29]. Model plant species are primarily used because their genomes are known, making the interpretation of findings simple and convenient; however, the fact that these are non-halophyte species means that these studies may not be suitable for the exploration of salt resistance mechanisms and the related genes.

With regard to studies on the responses and tolerance to salt, halophytes are ideal plants as they survive, grow normally and complete their life cycle in saline environments, some of which have salt concentrations equivalent to that of seawater. There are approximately 500 species of halophytes, constituting approximately 0.14% of known plant species [30]. However, the lack of whole-genome sequence data presents difficulties in considering these non-model plant species for use in understanding their response to salt stress at the molecular level. Nevertheless, high-throughput transcriptome sequencing, which is a powerful approach for discovering the molecular basis behind various biological events, is increasingly being used to understand the response to abiotic stresses at the genetic level. With regard to salinity, comparative transcriptome analyses have recently been reported for a few halophytes, such as *Sporobolus virginicus* [31], *Porteresia coarctata* [32], *Kosteletzkya virginica* [19], *Salicornia europaea* [33], *Mesembryanthemum crystallinum* [34], *Suaeda fruticosa* [35], *Zostera marina* [36] and *Halogeton glomeratus* [37], exposed and unexposed to salt.

The current work examines the response of *Suaeda maritima* (L.) to salt using high-throughput Illumina sequencing. This species is an herbaceous plant with succulent leaves that grows up to 30 cm in height and plays important roles in environmental protection, such as sand dune fixation. This species is a self-pollinated bisexual dicot of the family Chenopodiaceae that produces 1- to 2-mm reddish brown seeds, with *2n* = 36 chromosomes [38]. The optimal NaCl concentration for its growth is ~350 mM, but it grows well at a range of 500 mM to 50 mM NaCl, which makes it well-suited for studies on the response to salt [13] and for the physiological and molecular characterization of salt tolerance in plants [39]. Previously, Sahu and Shaw [13] reported it to be a glycinebetaine accumulator. The present work not only supports this finding but also reports numerous changes in gene expression in this plant in response to NaCl. This study identified many genes involved in biochemical and physiological processes, such as ion transport, osmolyte accumulation, cell wall remodeling, protein modification and degradation, antioxidative defense, and, more importantly, those involved in G protein and phosphoinositide signaling, which could be important for salt tolerance in plants.

## Results

### Transcriptome sequencing and assembly

After library construction using a Total RNA TruSeq kit (Illumina USA), RNA sequencing was performed on a HiSeq 2000 system using 100 * 2 paired-end sequencing chemistry technique. Output was generated as bcl files that were subjected de-multiplexing to convert them into fastq files. Samples were separated based on the barcodes/index values used in the library construction. The reads having ≥70% of the bases with a quality score ≥Q20 were chosen for downstream application. A quality distribution graph for the control and treated samples for both paired-end reads R1 and R2 depicting the maximum reads with Phred scores above 20 is shown in the S1 File. Deep sequencing to a coverage depth of 120x and 80x for control and treatment samples, respectively, using the Illumina platform yielded a total of 180,443,374 and 123,067,034 reads of 100 bases from the cDNA libraries prepared for the transcriptome sequencing of control and 2.0% NaCl-treated (9 h) *S*. *maritima*, respectively (Table 1). Low-quality reads were removed using the NGS (next generation sequencing) QC Toolkit, which finally yielded 178,620,034 (98.99%) and 121,809,974 (98.98%) high-quality (HQ) reads from the libraries of the control and treated plants, respectively (Table 1). The primary assembly provided 238,306 transcripts with an NP50 of 1,371 bp (Table 2). The NP50 value improved to 1,386 after running CD-HIT EST, and the total number of transcripts was reduced by 31% to 72,588 (Table 3). The authenticity of the transcript assembly was validated by aligning the reads from both the control and treated samples to all 72,588 transcripts using Bowtie 2. The total length of the de-novo assembled transcripts, which is a rough representation of total transcriptome size, indicates an approximate transcriptome size of 150 MB for the plant (Table 1).

**Table 1:** Details of the raw data and the quality control used for the assembly of the control and 2.0% NaCl-treated (9 h) *S*. *maritima* libraries.

Sample	Total paired raw reads	Total paired HQ reads	Low quality reads	Total data generated (GB)	Approximate transcriptome size (MB)	Expected sequencing depth
Control	180443374	178620034 (98.99%)	1823340	18.04	150	~120x
Treated	123067034	121809974 (98.98%)	1257060	12.31	150	~80x

**Table 2:** Primary assembly statistics of the control and 2.0% NaCl-treated (9 h) *S*. *maritima* libraries generated using Trinity assembler.

Parameters	Value
Total number of transcripts	238306
Transcriptome length (bp)	154678888
Minimum transcript length (bp)	201
Maximum transcript length (bp)	16589
Average transcript length (bp)	649.08
NP50 contig size (bp)	1371
(G+C)%	40.76

**Table 3:** Secondary assembly statistics of the control and 2.0% NaCl-treated (9 h) *S*. *maritima* libraries following CD-HIT EST analysis.

Parameters	Value
Number of clustered transcripts	72588
Clustered transcriptome length (bp)	64382019
Minimum transcript length (bp)	301
Maximum transcript length (bp)	16589
Average transcript length (bp)	886.95
NP50	1386
(G+C)%	40.34

### Functional annotation

The transcripts assembled were annotated by mapping the non-redundant sequences to the protein sequences available in PlantGDB (http://www.plantgdb.org) and the NCBI (http://www.ncbi.nlm.nih.gov) nr protein database using BLASTX searches. In the analysis, 27,434 (38%) transcripts out of 72,588 were annotated according to BLASTX scores using an e value cutoff of 1e-3 and with a minimum query coverage of 50% (S2 File). Several of the 27,434 ESTs identified hit the same protein in the BLASTX search, and hence, these were ultimately mapped to 19,850 unique genes (S2 File). These identified ESTs were found to match to 249 plant species (S2 File). Among these, the top ten plant species shared 87% similarity, while 13% similarity was shared by the remaining 239 plant species. The maximum similarity was shared with *Arabidopsis thaliana* (35.76%), followed by that with *Oryza sativa* (20.48%) and Zea mays (11.22%) (S3 File).

### Differential gene expression in response to salt application

Calculations of ΔΔCt values for the individual genes based on the ΔCt values of the control and treated samples obtained based on their RPKM (reads per kilo base per million) values revealed that 647 genes were upregulated and 735 genes were downregulated by at least 2-fold in response to NaCl application (S4 File). Moreover, there were 1,336 genes and 67 genes that were only expressed in either the control or the NaCl treated plants, respectively (S4 File). The top 20 upregulated genes and the top 20 downregulated genes are shown in the S5 File. The greatest upregulation noted was 7.52-fold for the gene encoding the serpin-Z12-like protein, and the greatest downregulation noted was 2.52-fold for the gene encoding cysteine-rich receptor-like protein kinase 6. The important genes among the top 20 that were upregulated that related to growth, development and salt tolerance were brassinosteroid hydroxylase, the sec14p-like phosphatidylinositol transfer family protein, protein phosphatase 2c, a cell surface glycoprotein, beta expansin and ABC transporter G family member 7. Moreover, there was an upregulation of the enzyme laccase 5, which is involved in cell wall lignification, and of a structural protein, extensin, which is also associated with cell wall maintenance. The top 20 genes that were downregulated included those that encoded proteins such as cysteine-rich receptor-like protein kinase 6, the ankyrin repeat-containing protein, NAC domain transcription factor superfamily proteins, a protein kinase, a serine protease (SBT2 protein), calmodulin binding protein, a leucine-rich repeat-containing protein, three receptor kinases and the hop-interacting protein THI116, which regulates important biochemical and molecular processes (S5 File). Heat maps generated to show the differential expression of the genes from various categories involved in important biochemical regulatory roles, including the genes encoding transcription factors (S6 File), proteins involved in ubiquitin-mediated modifications of proteins and their degradation (S7 File), enzymes and proteins facilitating the synthesis of hormones and osmolytes (S8 File), molecular chaperons facilitating protein folding and stabilization of the secondary structure of proteins (S9 File) and proteins involved in post-transcriptional modifications of mRNAs (S10 File), mostly showed that they were upregulated in response to NaCl treatment of *S*. *maritima*.

### Gene Ontology (GO) categorization of the total and differentially expressed genes (DEGs)

**Fig 1:**
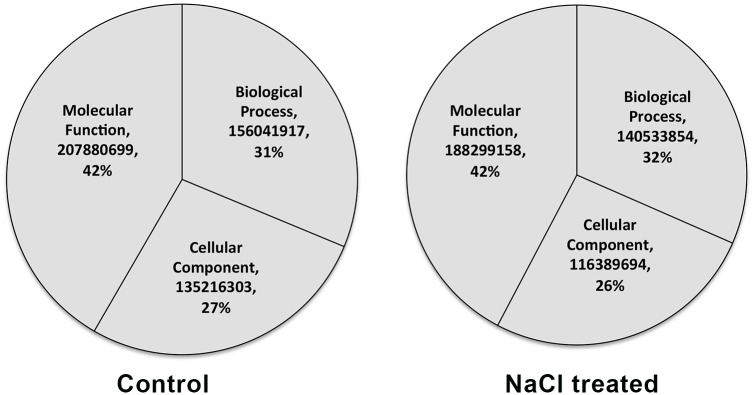
Pie chart showing the influence of NaCl on the distribution of reads among the three GO components, i.e., biological process, molecular function and cellular component.

GO categorization for all genes was performed based on the number of reads of the individual genes in the three main GO categories, i.e., biological process, molecular function and cellular component. There was no change in the percentage of reads in the GO enrichment for molecular function in response to salt application to *S*. *maritima* (Fig 1). The number of reads for the total genes in the biological process category increased by 1% and that in the cellular component category decreased by 1% in response to salt application.

**Fig 2:**
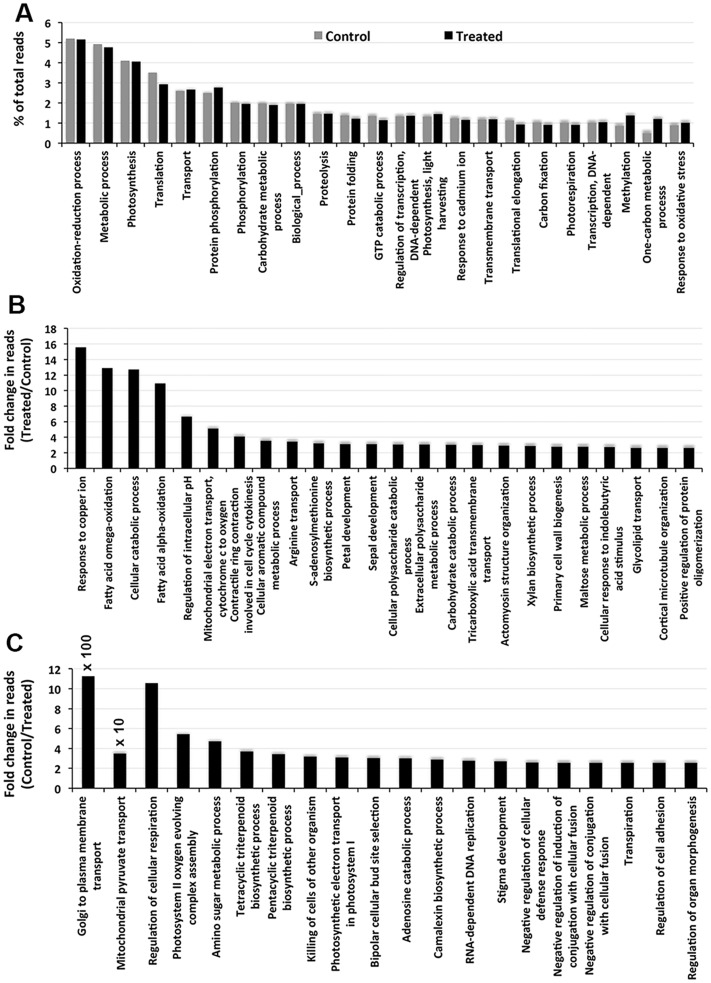
Top 20 Biological processes as revealed by the GO enrichment of the transcripts. A) The individual bars represent the percent contribution of the reads to the individual GO terms in decreasing order in the control and NaCl-treated plants. The individual bars in B) and C) represent increases and decreases, respectively, in the expression of the individual genes in the biological process categories presented as changes in the read values (fold changes) in decreasing order in the plants following NaCl application.

Only minimal differences were observed between the reads from the control and treated groups in terms of the percentage of the total reads for the genes categorized in the top 20 biological processes (Fig 2A). However, the expression of the genes related to translation, protein folding, GTP catabolic processes and translational elongation was decreased and that of genes related to protein phosphorylation, methylation and one-carbon metabolic processes was increased noticeably in response to salt application. The genes showing maximum upregulation or downregulation, however, did not belong to the GO categories of the constitutively highly expressed genes. This is reflected in Fig 2B and 2C, which represent the GO enriched categories of the top 20 upregulated and top 20 downregulated genes, respectively, in the biological process category. Those showing more than a 5-fold upregulation upon NaCl treatment belonged to categories such as response to copper ions, fatty acid omega oxidation, cellular catabolic processes, fatty acid alpha-oxidation, regulation of intracellular pH and mitochondrial electron transport (Fig 2B). Among the other genes, the upregulation of nine was 3-fold to less than 5-fold, and the remaining genes showed upregulations of less than 3-fold to more than 2.5-fold. The downregulation of genes upon NaCl application was more prominent than their upregulation (Fig 2C). The maximum downregulation observed was 1125.16-fold for a gene enriched in the GO category Golgi to plasma membrane transport. A second gene, belonging to the GO category mitochondrial pyruvate transport, was downregulated by as much as 34.86-fold. Moreover, two genes, one belonging to the GO category regulation of cellular respiration and the other to photosystem II oxygen complex assembly were downregulated by more than 5-fold. Among the remaining genes, 6 showed downregulation by more than 3-fold and the others by more than 2.5-fold.

**Fig 3:**
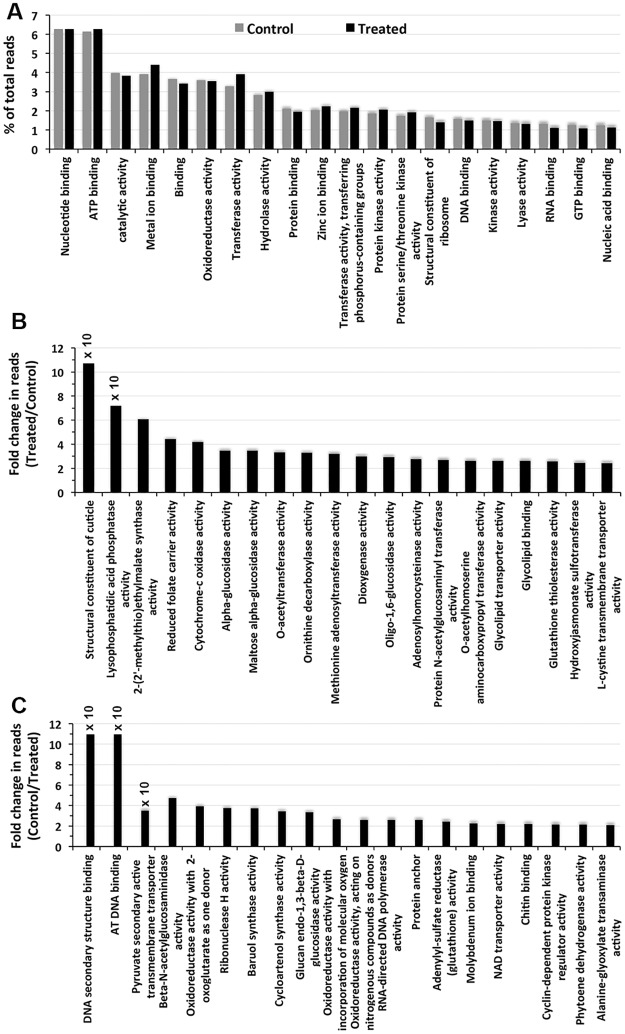
Top 20 Molecular function categories as revealed by the GO enrichment of the transcripts. A) The individual bars represent the percent contribution of the reads to the individual GO terms in decreasing order in the control and NaCl-treated plants. The individual bars in B) and C) represent increases and decreases, respectively, in the expression of the individual genes in the molecular function categories presented as changes in the read values (fold changes) in decreasing order in the plants following NaCl application.

Similar to the findings for the biological process category, the genes categorized in the top 20 molecular function GO categories did not show marked differences between the control and NaCl-treated plants (Fig 3A). However, there was a noticeable increase in the expression of genes in the metal ion binding and transferase activity GO categories. In contrast, the genes in the protein folding, structural constituent of ribosomes and RNA binding GO categories showed noticeable decreases upon salt application. Two genes individually enriched to the structural constituent of cuticles and lysophosphatidic acid phosphatase activity GO categories showed more than 70-fold increases in expression in response to the salt application (Fig 3B). Eight of the remaining top 20 genes that were upregulated showed increases in expression from 3.2-fold to 6.08-fold in response to NaCl treatment. The upregulation of the rest of the genes was more than 2-fold. Similar to the patterns of upregulation, the downregulation of at least three genes individually enriched in the DNA secondary structure binding, AT DNA binding and pyruvate secondary active transmembrane transporter activity GO categories was highly significant; the genes of the first two categories were downregulated by more than 100-fold, and the gene belonging to the third category was downregulated by more than 35-fold in response to the salt treatment (Fig 3C). The genes in the remaining GO categories showed downregulations from 2.0-fold to 4.73-fold in response to NaCl treatment.

**Fig 4:**
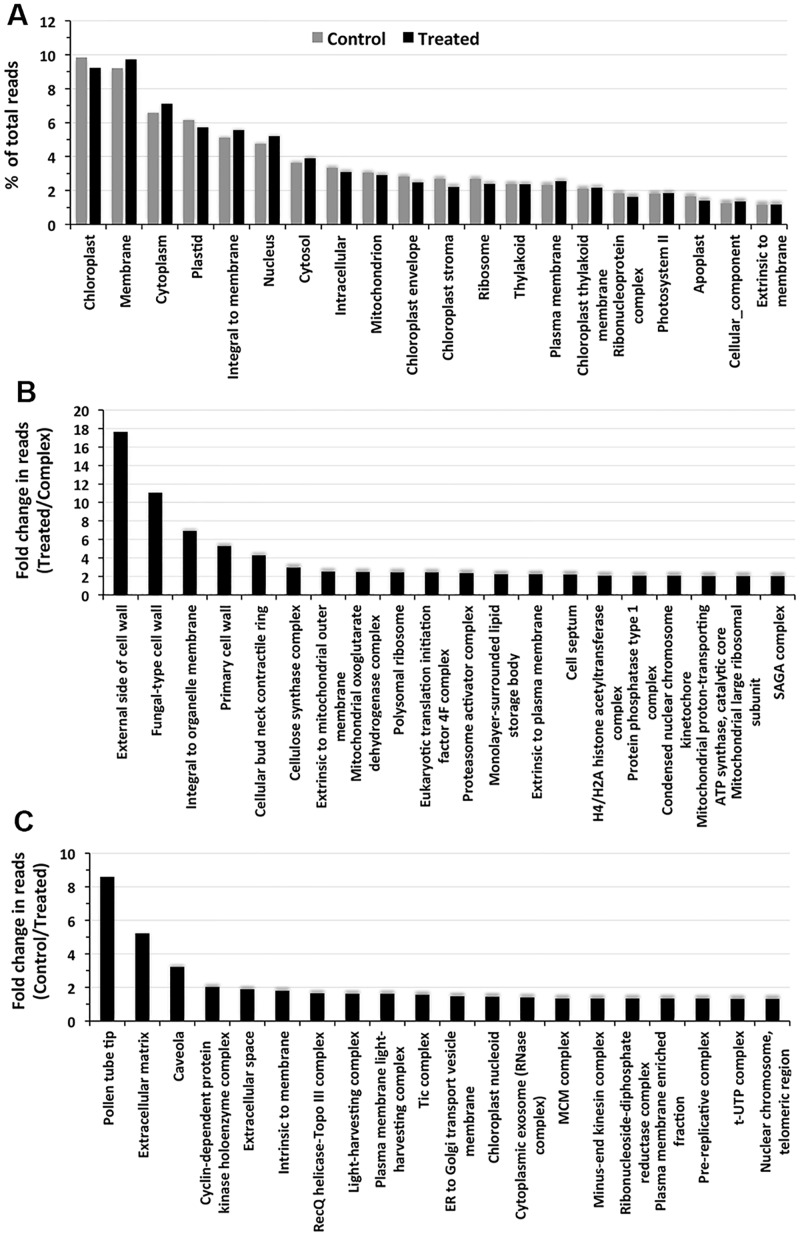
Top 20 Cellular component categories as revealed by the GO enrichment of the transcripts. A) The individual bars represent the percent contribution of the reads to the individual GO terms in decreasing order in the control and NaCl-treated plants. The individual bars in B) and C) represent increases and decreases, respectively, in the expression of the individual genes in the cellular components categories presented as changes in the read values (fold changes) in decreasing order in the plants following NaCl application.

Most of the highly expressed genes in the cellular component category enriched to the chloroplast, membrane, cytoplasm, plastid, integral to membrane and nucleus GO categories, representing more than 40% of the total reads (Fig 4A). Unlike the results in the biological process and molecular function categories, the differential expression of the genes under the individual cellular component GO categories was more noticeable, particularly for the genes enriched under the membrane, cytoplasm, integral to membrane and nucleus GO categories, where expression increased in response to NaCl application (Fig 4A). A noticeable downregulation in expression was observed for the genes enriched in the chloroplast, plastid and chloroplast stroma GO categories (Fig 4A). The top 20 upregulated genes showed variable responses, ranging from 2.02-fold to 17.64-fold, following NaCl application (Fig 4B). However, only four genes, enriching individually to the external side of cell wall, fungal-type cell wall, integral to organelle membrane, primary cell wall and cellular bud neck contractile ring GO categories, showed increases in expression of more than 3-fold (Fig 4B). In contrast to the changes in the upregulated genes, the downregulation of the genes enriched in the cellular component categories was not as pronounced (Fig 4C). The maximum gene downregulation, 8.59-fold, was observed for a gene enriched in the pollen tube tip GO category. The downregulation of three other genes that enriched individually to the extracellular matrix, caveola and cyclin dependent protein GO categories varied from 2.03-fold to 5.23-fold. The downregulation of the remaining genes in response to NaCl application was less than 2.0-fold.

### COGs and MapMan functional categorization of the DEGs

**Fig 5:**
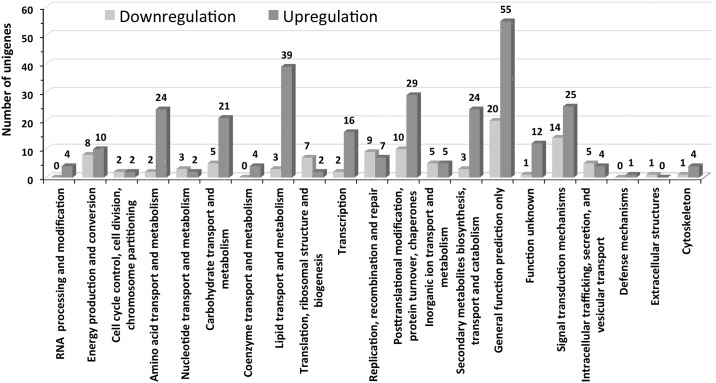
Functional categorization of unigenes based on the COGs database. The individual bars represent the number of unigenes that were downregulated or upregulated in each functional category in response to NaCl application.

Out of the more than 1500 genes showing a 2-fold change in expression following salt treatment, 391 proteins were found to be homologous to proteins in the COGs (cluster of orthologous groups) database and could be assigned to 21 functional categories (Fig 5). Most of the proteins were assigned to the following functional categories: energy production and conversion, amino acid transport and metabolism, carbohydrate transport and metabolism, lipid transport and metabolism, transcription, replication, recombination and repair, posttranslational modification, protein turnover and chaperons, secondary metabolite biosynthesis, transport and catabolism, general function prediction only, signal transduction mechanisms and proteins of unknown function. Among these, the majority of the proteins were grouped into the general function prediction only category, which is poorly characterized. In most of the categories, gene upregulation was more likely than downregulation in response to the salt treatment. The majority of the genes encoding proteins belonging to the amino acid transport and metabolism, carbohydrate transport and metabolism, lipid transport and metabolism, and secondary metabolite biosynthesis, transport and catabolism categories showed salt-induced upregulation. For the genes encoding proteins in the general function prediction only category, 55 out of 75 showed an upregulation in response to the salt treatment. A high number of genes encoding proteins categorized to the posttranslational modification, protein turnover and chaperones, and signal transduction mechanisms functional categories also showed upregulation. Moreover, in at least in two functional categories, RNA processing and modification, and co-enzyme transport and metabolism, additional genes were expressed.

**Fig 6:**
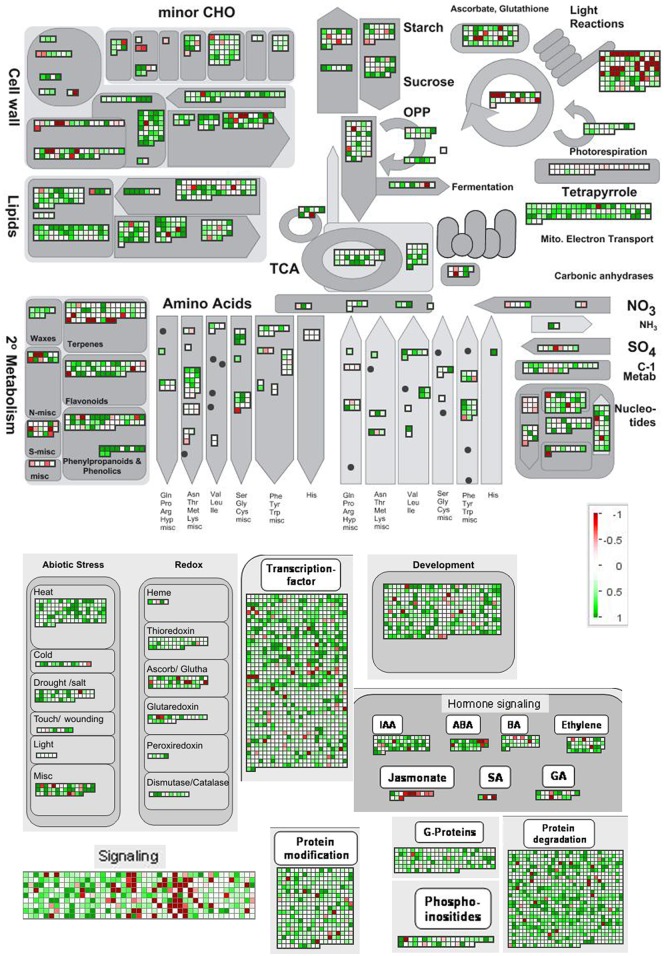
MapMan depiction of the unigenes expressed in control and NaCl-treated *S*. *maritima* plants related to different pathways. Each square represents individual genes of a pathway or functional category. The red and green squares represent decreases and increases, respectively, in the expression of a gene in response to NaCl application, presented as the fold change compared with expression in the control.

To further explore the influence of salinity on the metabolic processes of this species, all the genes were mapped using the MapMan tool (Fig 6). Table 4 presents the comparative details of the TAIR (The Arabidopsis Information Resource) genes assigned to various BINs, or functional categories, and their homologs present in *S*. *maritima*. Approximately 50% of the genes assigned to MapMan pathways showed homology to *S*. *maritima* (Table 4). The majority of the genes represent transcription factors (Fig 6). The next highest association was for genes involved in protein degradation, development and signaling. A large number of genes were also associated with lipid metabolism, cell wall synthesis and modification, and secondary metabolite synthesis, including that of flavonoids, phenolics and the osmoticum betaine. Moreover, the genes identified also contribute to both anabolic and catabolic carbohydrate metabolism, abiotic stress and redox responses, and G protein, phosphoinositide and hormone signaling.

**Table 4:** BINs/functional categories of the MapMan pathways representing the TAIR genes assigned to each BIN, the homologs found in *S*. *maritima* and their up- and down-regulation in response to NaCl application. Upregulation and downregulation represent 2-fold or greater change in expression of the gene and/or a change in expression significant at *p* ≤ 0.05.

BIN ID	BIN Name	Assigned Genes (TAIR)	Homologs in *S*. *maritima*	Up	Down
1	Photosynthesis	657	391	3	30
2	Major carbohydrates	379	238	4	5
3	Minor carbohydrates	320	173	5	4
4	Glycolysis	138	71	0	4
5	Fermentation	40	26	0	1
6	Gluconeogenesis/glyoxylate cycle	17	11	0	1
7	OPP cycle	56	30	1	0
8	TCA/organic acid transformations	143	96	1	2
9	Mitochondrial electron transport / ATP synthesis	319	165	4	3
10	Cell wall	1185	538	12	12
11	Lipid metabolism	1105	549	14	14
12	Nitrogen metabolism	89	49	4	3
13	Amino acid metabolism	684	417	6	11
14	Sulphur assimilation	16	10	0	1
15	Metal handling	178	85	2	1
16	Secondary metabolism	1235	563	22	28
17	Hormones	1922	903	22	38
18	Co-factor and vitamin metabolism	172	117	1	3
19	Tetrapyrrole synthesis	249	154	2	5
20	Stress: biotic and abiotic response	2648	1078	40	52
21	Redox	635	298	4	9
22	Polyamine	43	23	1	2
23	Nucleotide metabolism	302	180	1	7
24	Biodegradation of Xenobiotics	49	27	1	2
25	C1-metabolism	55	35	0	2
26	Miscellaneous enzyme families	3681	1626	32	60
27	RNA: processing, transcription, regulation of transcription and binding	7109	3402	39	41
28	DNA: synthesis/chromatin structure, repair and unspecified	3943	692	7	13
29	Protein: amino acid activation, synthesis, targeting, post-translational modification degradation, folding, glycosylation and assembly	9039	4229	47	98
30	Signaling	3304	1627	23	99
31	Cell: organization, division, cycle and vesicle transport	1487	836	11	15
33	Development: Storage proteins, inhibitors and unspecified	1937	812	25	11
34	Transporters	2274	1206	18	25
35	No ontology, Hypothetical or unknown proteins	7476	1830	32	23

The color codes indicate that the genes assigned to most of the metabolic pathways showed upregulation in *S*. *maritima* upon the NaCl treatment (Fig 6). The most significant influence was observed on the genes encoding transcription factors and those involved in protein degradation, development and protein modification. Most of the genes concerned with lipid metabolism and cell wall synthesis and modifications also showed upregulation in response to the salt treatment. The next most significant influence of the salt treatment was the increase in the expression of the genes associated with hormone signaling and the synthesis of the secondary metabolites, including flavonoids, phenolics and the osmoticum betaine. The genes involved in one-carbon compound metabolism and the metabolism of amino acids and nucleotides also showed enhanced expression in response to the salt treatment. Another important effect of the salt treatment relevant to plant life was the increase in the expression of the genes involved in the synthesis of tetrapyrroles, precursors of chlorophyll biosynthesis. However, in contrast, most of the genes encoding proteins involved in the light reactions were downregulated in response to the salt application. Concomitantly, many genes encoding proteins/enzymes of the Calvin cycle showed a downregulation in response to the salt application. The expression of most of the genes of the anabolic pathways, such as sucrose and starch synthesis, was enhanced upon the treatment of plants with NaCl. The genes involved in the synthesis of amino acids also showed increased expression in response to NaCl treatment. Most of the DEGs of catabolic pathways, such as the tricarboxylic acid cycle, glycolysis and the oxidative pentose phosphate pathway, also showed increased expression in response to the salt treatment, similar to the patterns observed for the genes of the anabolic pathways.

However, among the genes found to be homologous to the MapMan pathway, a much smaller fraction showed a significant upregulation or downregulation in response to the salt treatment (Table 4, S11 and S12 Files); only 612 genes assigned to the MapMan metabolic pathways showed 2-fold or greater changes in expression and/or significant differential expression at *p* ≤ 0.05. These were grouped into 34 BINs/categories (Table 4). Many of the BINs showed a strong response to salt. For the genes associated with signaling, including hormone signaling and protein modification and degradation, the number showing a significant downregulation was much higher than the number showing a significant upregulation (Table 4, S11 File). In contrast, the genes associated with development were much more likely to be significantly upregulated than they were to be significantly downregulated in response to the salt treatment. The genes associated with most of the other metabolic pathways, including those associated with cell walls, lipid metabolism, secondary metabolism, stress responses and RNA processing, showed more or less similar levels of significant down- and up-regulation.

### Response of ion transport components to NaCl application

**Fig 7:**
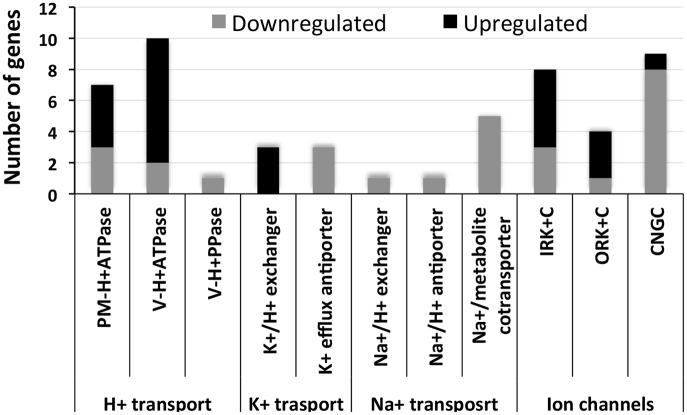
Proteins/enzymes involved in ion transportation in *S*. *maritima*. Each stacked column represents the number of isoforms of the proteins/enzymes downregulated (grey) and upregulated (black) in the plants in response to NaCl treatment.

NaCl application resulted in both the upregulation and downregulation of the genes encoding proteins and enzymes involved in ion transport (Fig 7). The response was isoform specific for *PM-H*^*+*^*ATPase* (plasma membrane H^+^ATPase), *V-H*^*+*^*ATPase* (vacuolar H^+^ATPase), *IRK*^*+*^*C* (inward rectifying K^+^ channel), *ORK*^*+*^*C* (outward rectifying K^+^ channel) and *CNGC* (cyclic nucleotide-gated channel). Four of the seven isoforms of *PM-H*^*+*^*ATPase* were upregulated and three were downregulated, while eight of the ten isoforms of *V-H*^*+*^*ATPase* were upregulated in response to NaCl application and two were downregulated. Among the ion channels, both *IRK*^*+*^*C* and *ORK*^*+*^*C* showed a greater number of the isoforms becoming upregulated than those that were downregulated in response to NaCl application. Only CNGC showed a greater number of its isoforms being downregulated than being upregulated. Among the *V-H*^*+*^*PPase* (vacuolar H^+^ pyrophosphatase), K^+^/H^+^ exchanger, K^+^ efflux antiporter, Na^+^/H^+^ exchanger, Na^+^/H^+^ antiporter and Na^+^/metabolite cotransporter, only the K^+^/H^+^ exchanger was upregulated, while the others were downregulated.

### Validation of the result of deep sequencing by qPCR

**Fig 8:**
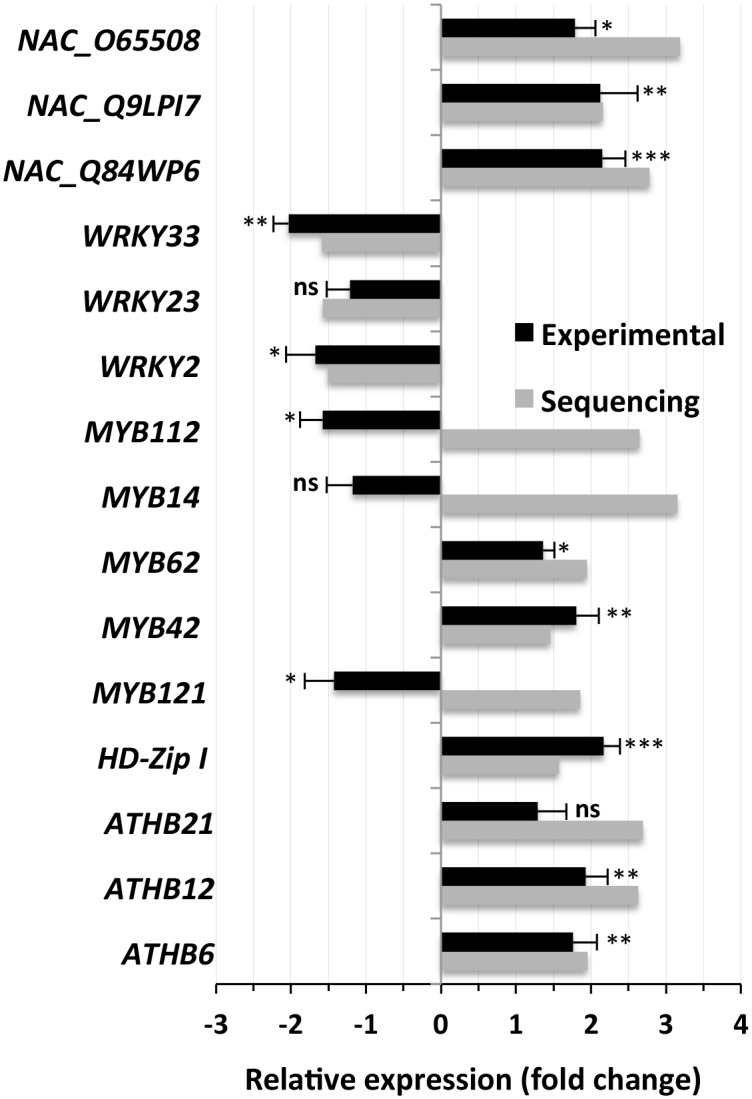
Changes in the expression of transcription factors in *S*. *maritima* in response to NaCl application. The grey bars represent the transcription factors that showed significant changes (2-fold or greater and/or changes significant at *p* ≤ 0.05), either upregulation or downregulation, based on the sequencing results in response to NaCl application. The corresponding black bars show expression levels as determined by qPCR, expressed as fold-changes relative to expression in the controls, in response to the NaCl application. The individual black bars, representing the qPCR data, are the means ± SD of six measurements (three technical replicates each for two biological samples). *, ** and *** next to a column indicate significant changes in expression at *p* ≤ 0.05, *p* ≤ 0.01 and *p* ≤ 0.001, respectively. Ns = not significant.

The expression results of the deep sequencing of a few groups of genes involved in various biochemical pathways and physiological functions related to the abiotic stress response were considered for validation by real-time PCR (qPCR). Several transcription factors were also selected for the validation of their expression, mainly those that showed significant changes in expression in the deep sequencing results in response to NaCl application (S4 File). qPCR results showed changes in expression that were similar to the NGS results for most transcription factors (Fig 8). Only the qPCR validations of *MYB112* (MYB, myeloblastosis), *MYB14* and *MYB121* showed results opposite to those from the deep sequencing. The changes in expression of most of the transcription factors studied by qPCR were significant, except for those of *WRKY23*, *MYB14* and *ATHB21* (ATBH, *Arabidopsis thaliana* homeobox). The maximum upregulation in response to salt application observed by qPCR was for *NAC_Q84WP6*.

**Fig 9:**
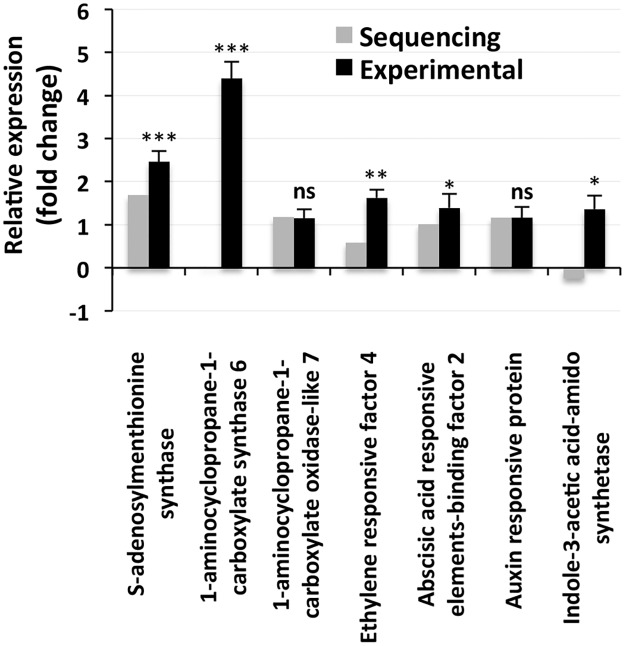
Relative expression of the proteins/enzymes involved in plant hormone synthesis. The grey bars represent the sequencing results, while the corresponding black bars represent the results that were obtained by qPCR. The individual black bars, representing the qPCR data, are the means ± SD of six measurements (three technical replicates each for two biological samples). *, ** and *** next to a column indicate significant changes in expression at *p* ≤ 0.05, p ≤ 0.01 and *p* ≤ 0.001, respectively. Ns = not significant.

**Fig 10:**
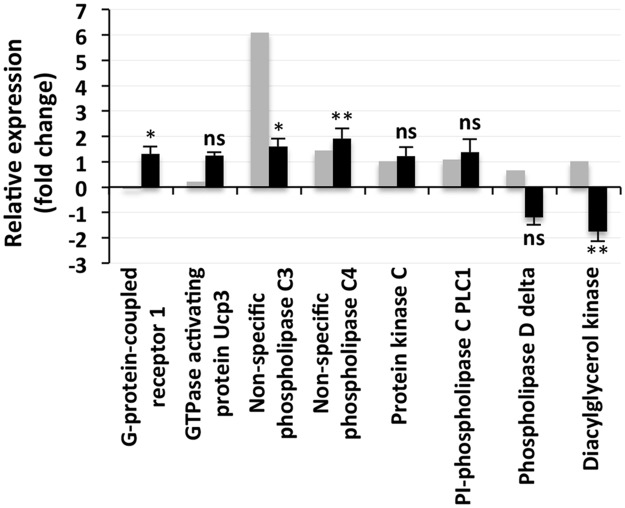
Relative expression of the proteins/enzymes involved in G protein and phosphoinositide signaling. The grey bars represent the sequencing results, while the corresponding black bars represent the results that were obtained by qPCR. The individual black bars, representing the qPCR data, are the means ± SD of six determinations (three technical replicates each for two biological samples). * and ** next to a column indicate significant changes in expression at *p* ≤ 0.05 and *p* ≤ 0.01, respectively. Ns = not significant.

All the genes involved in hormone biosynthesis and hormonal responses that showed increases in expression in the deep sequencing results following salt treatment also showed enhanced expression in the qPCR analysis (Fig 9). Moreover, the qPCR results showed an increase in the expression of 1-aminocyclopropane-1-carboxylate synthase (*ACS*) and indole-3-acetic acid-amido (*IAA-AM*) synthase in response to NaCl application, which is in contrast to the downregulation of these genes found in the NGS results. The qPCR analysis also revealed significant increases in the expression of most of the genes involved in G protein and phosphoinositide signaling (Fig 10), similar to the patterns shown for hormone synthesis and hormonal responses. Genes for two proteins, phospholipase D (*PLD*) delta and diacylglycerol kinase (*DGK*), involved in phosphoinositide signaling were found to be downregulated in the qPCR analysis, which is in contrast to the results obtained with deep sequencing (Fig 10). However, the downregulation was only significant for *DGK*.

**Fig 11:**
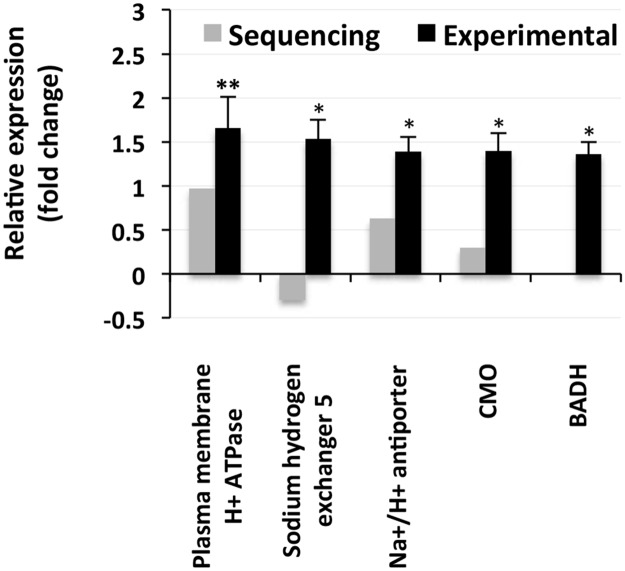
Relative expression of the proteins/enzymes involved in Na^+^ transport and sequestration and glycinebetaine accumulation. The grey bars represent the sequencing results, while the corresponding black bars represent the results that were obtained by qPCR. Sequencing expression data were not available for *BADH*. The *BADH* sequence of *S*. *maritima* was retrieved from the primary assembly sequence data via a similarity search of the forward primer used for the PCR amplification of the *BADH* gene from *Suaeda salsa* [13]. The individual black bars, representing the qPCR data, are the means ± SD of six measurements (three technical replicates each for two biological samples). * and ** next to a column indicate significant changes in expression at *p* ≤ 0.05 and *p* ≤ 0.01 levels, respectively.

All three genes involved in ionic adjustments that were selected for expression validation by qPCR showed a significant upregulation in response to the salt treatment (Fig 11). Among these, the gene encoding a sodium hydrogen exchanger, showed a downregulation in the deep sequencing results. Both the genes involved in glycinebetaine synthesis, namely, betaine aldehyde dehydrogenase (*BADH*) and choline monooxygenase (*CMO*), exhibited significant increases in expression in the plants in response to the salt treatment. NGS data also revealed an increase in the expression of *CMO*. However, the *BADH* sequence was not available in the secondary assembly NGS data used for the statistical analysis, and hence, its sequencing expression results are not presented.

**Fig 12:**
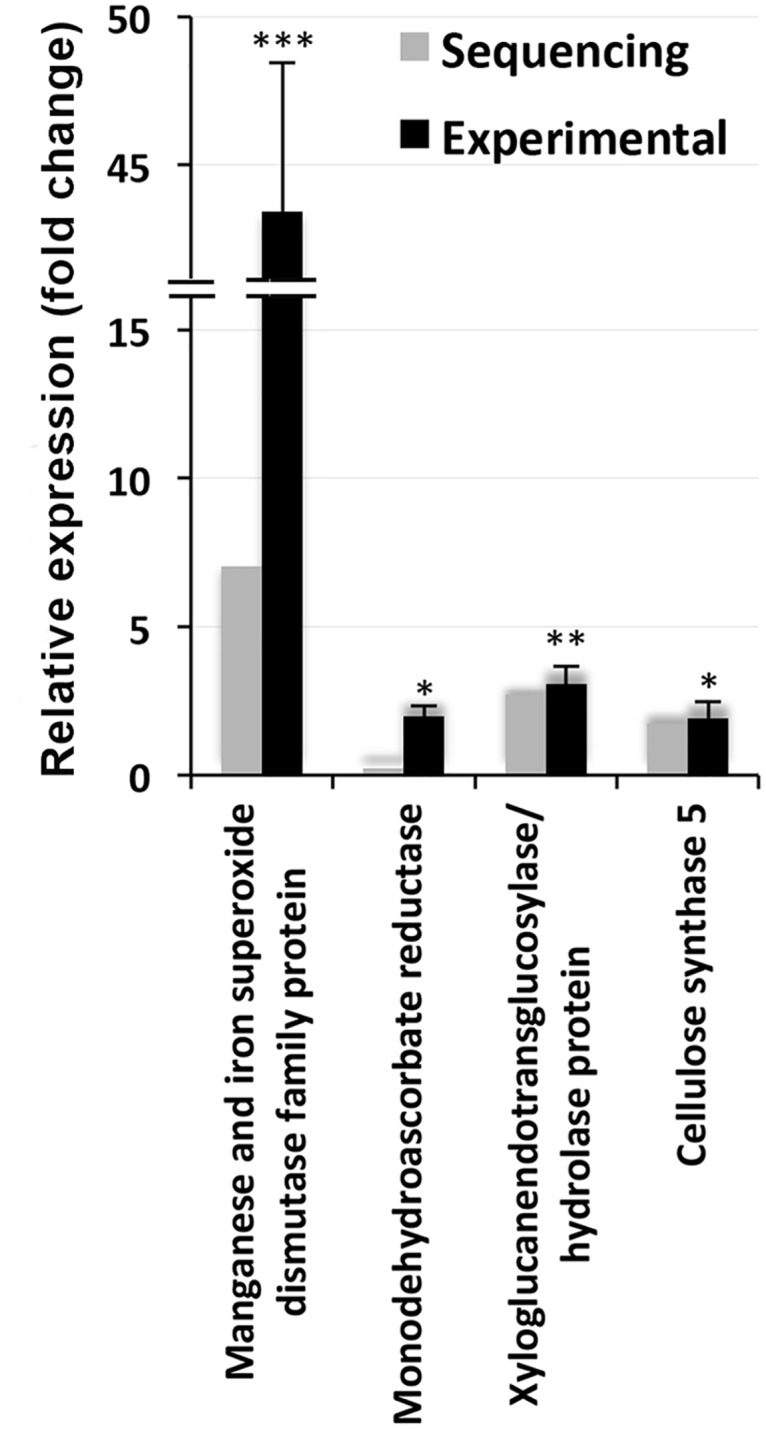
Relative expression of the proteins/enzymes involved in antioxidative defense and cell wall metabolism. The grey bars represent the sequencing results, while the corresponding black bars represent the results that were obtained by qPCR. The individual black bars, representing the qPCR data, are the means ± SD of six measurements (three technical replicates each for two biological samples). *, ** and *** next to a column indicate significant changes in expression at *p* ≤ 0.05, *p* ≤ 0.01 and *p* ≤ 0.001, respectively.

Among the other genes selected for the validation of their expression by qPCR, genes for the manganese and iron superoxide dismutase (SOD) family protein showed the greatest response, with a more than 40-fold increase in expression in response to the salt treatment, although the increase in expression in the deep sequencing results was only 7.04-fold (Fig 12). The expression of the other genes, including those for monodehydroascorbate reductase (*MDHAR*), the xyloglucan endotransglucosylase/hydrolase protein and cellulose synthase 5, were also found to be significantly increased in response to the salt treatment, in agreement with the increase in their expression observed in the deep sequencing results.

## Discussion

High-throughput sequencing is widely being used for the transcriptome profiling of plant species with unknown genomes to obtain information about the metabolism of the organisms that is specific to certain ecological conditions [40] or to understand the genetic relatedness between two or more species [36–37,41–43]. The technology is especially important in understanding the response of an organism to an environmental condition at the genetic level and in identifying the genes and/or the metabolic processes enabling the organism to tolerate extreme environments by comparative functional genomics between normal and extreme conditions [19,29,31,32–33,35,37,41–43]. The depth of sequencing required to obtain most of the information for a genome of a species depends on the size of the genome, and for an unknown genome, 10 GB of data for 50 million 100-base paired-end reads is considered to be sufficient. The high quality 100-base reads of ~178 and ~121 million obtained for the control and NaCl treated samples, respectively (Table 1), is therefore likely to represent most of the genome of *S*. *maritima*. The primary assembly with a total transcript number of 238,306 and the secondary assembly with a total clustered transcript number of 72,588 supports this conclusion as the data on the number of reads and the unigenes assembled are comparable with data from previous reports [29,31–32,35,37,42–43]. The NP50 value of the transcripts, i.e., the value represented by 50% of the transcripts, of both primary and secondary assemblies was greater than 1300 bp (Tables 2 and 3), suggesting that the sequencing results are reliable [29,31–32,35,37,42–43]. However, only 38% of the genes assembled were able to be matched to the database (S2 File), suggesting that much of the genome of *S*. *maritima* is unknown and differs greatly from that of the model plants, such as *Arabidopsis*, maize and rice, that are generally used in experiments (S3 File). Functional characterizations of novel genes without a homologous match in existing databases are needed to obtain a true picture of the processes involved in salt tolerance in *S*. *maritima*. An analysis of the DEGs in response to salt application revealed that the response of most of the genes might be a consequence of salt exposure rather than having any direct role in salt tolerance as none of the top twenty upregulated or downregulated genes (S5 File) have been reported to have any direct relation with salt tolerance.

The GO enrichment of individual genes to GO terms is widely used to characterize the influence of an abiotic stress on an organism [43–44]. However, the top twenty GO terms in the main GO categories showed little influence of the salt treatment on the expression of the genes enriched to these GO terms (Figs 2A, 3A and 4A), suggesting that genes constitutively expressed at high levels are not relevant to the environmental change and, hence, may not be of importance for salt stress tolerance. This is supported by the fact that the GO terms enriched by the genes expressed at low levels (based on the number of reads) in all the main GO categories revealed a significant up- or down-regulation of the read numbers in response to the salt treatment (Figs 2B, 2C, 3B, 3C, 4B and 4C). This observation suggests that it is the genes expressed at low levels that let *S*. *maritima* adapt to the changing environmental condition. In this context, it also makes sense that only a significant difference in the product of a gene is going to cause a significant change in plant metabolism that is important for tolerating changes in the environment. The GO terms reportedly influenced by salinity in *Gossypium aridum* and *Phaseolus vulgaris* [43–44] were, however, not identified in the current study, which is likely because these species are glycophytes, while *S*. *maritima* is a halophyte. Such GO analyses are scant in halophytes. The GO enrichment of the DEGs in this study revealed that salt tolerance must be an energy consuming process as one or more GO terms showing a salt-induced upregulation of the reads in all three main categories were linked to ATP generation, such as the mitochondrial electron transport cytochrome C to oxygen (Fig 2B), cytochrome C oxidase activity (Fig 3B), and the mitochondrial oxoglutarate dehydrogenase complex and mitochondrial proton transporting ATP synthase catalytic core (Fig 4B).

The homology of only 391 genes to the COGs database out of the ~1500 DEGs indicates that the genome of *S*. *maritima* is very different from those of the proteins represented in the COGs database, which has protein sequences from 66 genomes [41]. Reports on the alignment of differentially expressed proteins to the COGs database are not available, although the alignment of total proteins to the COGs database has been performed for a few halophytes, including *Salicornia europaea* [33] and *Avicennia marina* [40]. Moreover, the alignment of even the total protein of these halophytes to the COGs database is only ~16% [33,40], in contrast to the ~40% alignment shown by glycophytes [44]. The alignment of only 26% of the salt-induced differentially expressed proteins in *S*. *maritima* suggests that much of the information of salt tolerance is still hidden or unexplored. Among the identified proteins, those involved in 1) amino acid transport and metabolism, 2) lipid transport and metabolism, 3) post translational modification, protein turnover and chaperones, 4) secondary metabolite synthesis, transport and catabolism, and 5) signal transduction mechanisms might play important roles in salt tolerance as most of them were significantly upregulated in response to the salt treatment (Fig 5). Moreover, the salt-induced upregulation of many of the poorly characterized proteins (in the general function prediction only and function unknown categories) also indicates the possibility of their important roles in the salt tolerance process.

Compared with the 391 genes with identified COG homologies, 612 DEGs (downregulated/upregulated by 2-fold or more or significant changes in expression at p ≤ 0.05) were found to have homologies in the MapMan pathways, indicating MapMan to be an advanced bioinformatics tool for the comprehensive interpretation of transcriptome data and the visualization of the functions of the associated genes (Fig 6). This is further reflected by the fact that nearly 50% of the TAIR-assigned genes shared homologies with the genes expressed in *S*. *maritima* (Table 4). The usefulness of MapMan in the study of cellular responses to external stimuli or abiotic stress has been shown in a few studies [42,45]. The major impact of salt treatment in *S*. *maritima* seems to be on cell wall metabolism because the cell wall BIN showed significant changes in 24 genes (Table 4), and there were significant changes in the expression of 50 genes related to the products of secondary metabolism (Table 4), many of which are the components of cell walls. The responsiveness of cell wall metabolism genes to salt stress is in agreement with a report that the cell wall undergoes considerable restructuring in response to changes in salinity, both in salt-tolerant and non-tolerant genotypes [46], suggesting it to be highly dynamic component of plant cells [47]. Significant increases in the expression of cellulose synthase, extensin 1 and xyloglucan endotransglucosylase (S11 File) especially indicate an increase in cell wall synthesis activity. While cellulose synthase synthesizes the cellulose component of the cell wall, xyloglucan endotransglucosylase, which has been shown to have high activity at sites of cell elongation in both roots and shoots [48], modifies the xyloglucan-cellulose framework that facilitates the expansion of cell walls [49], and extensin provides the platform over which the self-assembly of carbohydrate components occur in the cell wall [50]. A qPCR analysis validated the salt-induced upregulation of cellulose synthase and xyloglucan endotransglucosylase (Fig 12). The salt-induced increase in cell wall synthesis activity shown here in *S*. *maritima* is in agreement with demonstrations of improved growth of this plant in the presence of salt relative to its growth without salt [51]. The salt-induced upregulation of the genes involved in phenylpropanoid and phenolic biosynthesis (Fig 6) indicates that the presence of salt also promotes the lignification of the cell walls that is essential for preventing pathogen invasion and reinforcing the cellulose wall structure for increased mechanical strength [52]. Moreover, the upregulation of the genes encoding enzymes, such as of laccase (S11 File) that is involved in the delignification of lignocellulosics [53] and of O-methyltransferases (S11 File), which play key roles in lignin biosynthesis [54], suggest the dynamic nature of cell wall restructuring for promoting and facilitating the growth of halophytes in presence of salt.

The genes involved in lipid metabolism showed the second most significant differential expression in response to salt application after those involved in cell wall and secondary metabolisms (Fig 6). The essential role of lipids at the cellular and molecular level in living organism is well established and is reflected in the fact that these molecules enable the formation of boundaries between cells and organelles, serve as rich sources of energy and act as signaling molecules. At least three genes shown to be upregulated in response to salt application, encoding fatty acid desaturase, alpha/beta hydrolase and lipid transfer proteins (S11 File), have been shown in transgenic experiments or mutation studies to provide salinity tolerance to plants [55–57], indicating the important role of these genes in salt tolerance in *S*. *maritima*. This plant also shows a salt-induced upregulation of serine decarboxylase, an enzyme unique to plants that is involved in the synthesis of ethanolamine [58]. Ethanolamine is utilized in the synthesis of phosphatidylethanolamine, phosphatidylcholine and choline. While the former two compounds form the major phospholipid constituent of eukaryotic membranes, choline forms the precursor for glycinebetaine synthesis [13], an osmoticum demonstrated to be accumulated in plants in response to drought and high salinity [59].

One of the noticeable impacts of salt application was the decrease in the expression of genes related to photosynthesis, particularly the light reaction (Fig 6, Table 4, S11 File). In contrast, the expression of the genes involved in the metabolism of tetrapyrrole, the active core of chlorophylls, increased in response to salt application, indicating increases in chlorophyll biosynthesis. This is in agreement with the greater photosynthetic efficiency observed in plants grown in the presence of salt compared with that of plants grown without salt [60]. An upregulation of the genes involved in the developmental process (Fig 6) also supports the idea of improved growth performance by *S*. *maritima* in the presence of salt. The downregulation of the expression of genes related to the light reactions observed in the present study might therefore be a short-term response.

The regulation of the turnover and modification of proteins seems to be an important aspects of salt tolerance in halophytes, as reflected by the upregulation of the genes involved in protein degradation and modification (Fig 6). Genes involved in ubiquitination-mediated degradation of proteins have been reported to be associated with responses to salt in halophytes [60]. In fact, the ubiquitin/26S proteasome system has been reported to be a positive regulator of stress tolerance in plants [61]. The role of phosphorylation in the modification of proteins for improving, regulating or determining their function is well established [62]. Moreover, proteomic studies have indicated possible important roles of the carbonylation and S-nitrosylation of proteins in salt tolerance in plants [63].

The upregulation of genes linked to metabolic responses to abiotic stress and the maintenance of redox status in response to salt application (Fig 6, Table 4) is an indication of a shift in the balance of the production of reactive oxygen intermediates (ROIs), such as superoxide radicals (O2^·-^) and H_2_O_2_, which have been reported to increase as a result of disturbances in metabolic activities in plants experiencing a change in their external environment [64]. Environmental factors such as drought, salinity, and heat have been reported to shift the balance towards an increase in the generation of ROIs, resulting in their accumulation in cells and tissues and, subsequently, oxidative damage [16,64]. The highly significant upregulation of manganese and iron superoxide dismutase proteins concomitant with a significant increase in the expression of *MDHAR* (Fig 12) and a more than 2-fold change in Cu/Zn-SOD expression (S11 File) in response to the salt application are all indicative of the activation of the antioxidant system of *S*. *maritima* for the effective removal of ROIs. These results thus suggest that the antioxidant system is also an important component of salt tolerance in plants [13,16].

Although the MapMan analysis identified a large number of transcription factors, both those that were up- and down-regulated in *S*. *maritima* in response to NaCl application (Fig 6), only a few showed significant changes in their expression (S11 File). The qPCR analysis largely validated the expression of the selected transcription factors (Fig 8), except of *MYB112*, *MYB14* and *MYB121*, indicating that the libraries prepared for deep sequencing were reliable. The significant upregulation of most of the members of the NAC transcription factor family in response to the salt application (S11 File, Fig 8) indicates their possible involvement in salt tolerance in halophytes. NAC constitutes a large family of transcription factors specific to plants [65]. The genome-wide identification of NAC proteins has been carried out in several plants [66–67], but not in halophytes. Moreover, several members of NAC transcription factors have been reported to be responsive to salt and other abiotic stresses in plants such as *A*. *thaliana*, *V*. *vinifera* and *P*. *pinaster* [28,66–67]. Many of the NAC transcription factors overexpressed in plants such as *Arabidopsis* and rice have also been reported to confer tolerance to salt and other abiotic stresses [28,65,68], indicating their great potential for improving salt tolerance in crop plants via biotechnological approaches. Hence, the NAC proteins identified to be upregulated in response to the salt application in this study could be of significance, particularly considering that the plant studied was a halophyte.

Similar to NAC, the MYB families of transcription factors show an early response to environmental stimuli [69], but unlike the NAC proteins, they are not plant-specific. A genome wide analysis has revealed the presence of 155 and 197 *MYB* genes in rice and *Arabidopsis*, respectively [70]. Genome wide expression profiling of these genes under abiotic stresses, including salinity, has also been carried out in rice and *Arabidopsis*, but the results are highly variable depending upon the test species and the cultivars used [70]. Nevertheless, the overexpression of certain *MYB* genes, such as *TaMYB73* and *AmMYB1*, in rice and tobacco, respectively, has been shown to increase the salt tolerance of transgenic plants [71–72]. Tolerance to other abiotic stresses, however, was also increased simultaneously, suggesting that *MYB* genes might not be specifically involved in salt tolerance. The significant salt-induced upregulation of the *MYB* genes in the current study (S11 File, Fig 8) also suggests their possible regulatory role in abiotic stress tolerance in plants, particularly as the results are similar to those reported for *AmMYB1* cloned from the halophyte *A*. *marina* [72].

Homeodomain-leucine zipper (HD-Zip) transcription factors, including homeobox 12 (ATHB12), ATHB6 and HD-Zip I, are unique to plants, as are the NAC transcription factors. These transcription factors have been reported to be involved in a wide range of physiological functions, including mediation of growth responses to water deficits [73], the regulation of floral development [74] and cell cycle regulation [75]. The upregulation of members of the *HD-Zip* transcription factor group in the present study in response to salt application also indicates their important role in abiotic stress tolerance. In fact, *ATHB12* has been reported to increase salt tolerance in yeast by regulating sodium exclusion [76], and the expression of *ATBH6* is upregulated in *Arabidopsis* under water deficit conditions [77].

The significant differential expression of other transcription factors in response to NaCl application was limited to the downregulation of *WRKY* (S11 File, Fig 8), suggesting the relative unimportance of these other groups in the processes related to salt tolerance in plants, especially when compared to importance of *NAC*, *MYB* and *HD-Zip*. *WRKY* has also been reported to be downregulated in the halophyte *Suaeda fruticosa* upon NaCl application [35]. Nevertheless, WRKY proteins have been reported to be responsive to drought and salinity [78]. Moreover, the overexpression of *AtWRKY25* and *AtWRKY33* has been shown to lead to enhanced salt tolerance in transgenic *Arabidopsis* [79]. These examples clearly indicate that WRKY transcriptional factors are associated with transcriptional reprogramming in plants challenged with abiotic stresses and, hence, are important in abiotic stress tolerance.

Although no significant upregulation of the genes related to Na^+^ transport was observed in the sequencing results (S11 File), several genes related to ion transport were upregulated in response to NaCl application (Fig 7), indicating their importance in salt tolerance. Moreover, the qPCR analyses of the *PM-H*^*+*^*ATPase*, sodium hydrogen exchanger and Na^+^/H^+^ antiporter showed that they were significantly upregulated in response to NaCl application (Fig 11), suggesting that they might be important for salt tolerance in plants. These proteins have previously been reported to be upregulated in plants upon salt treatment [14,31,36].

The significant increases in the expression of S-adenosylmethionine (*SAM*) synthase, *ACS*, *ACO* and ethylene responsive factor in response to NaCl application (Fig 9) indicate the important role of ethylene in salt tolerance in *S*. *maritima* in addition to the roles of transcription factors and the genes involved in ion transportation. The increase in the expression of *SAM* synthase in the current study is in agreement with that reported by Sahu and Shaw [13], who also related it to the biosynthesis and accumulation of glycinebetaine, an osmoticum, in *S*. *maritima* via ethanolamine synthesis in addition to the requirement of SAM in ethylene synthesis. A significant upregulation of *CMO* and *BADH* in response to the salt treatment (Fig 11) supports the findings of Sahu and Shaw [13] and Gharat and Shaw [51] that *S*. *maritima* is a betaine accumulator. Moreover, Sahu and Shaw [13] also reported an increase in the expression of ethylene responsive element binding protein (*EREBP*) in *S*. *maritima* in response to salt treatment, which is similar to the findings of this study, further suggesting the possible involvement of ethylene and ethylene responsive factors (ERFs) in salt tolerance in halophytes. In contrast, the response of *ERFs* to salt is contradictory in glycophytes; while salt treatment leads to an increase in the expression of *ERFs* in rice [80], it downregulates their expression in *Arabidopsis* [81]. Similar to that of ethylene, the salt-induced expression of ABA responsive element-binding factor (*ABAREBF*) (Fig 9), auxin responsive protein and *IAA-AM* synthetase is suggestive of the possible involvement of auxin (indole-3-acetic acid, IAA) and ABA in salt tolerance in *S*. *maritima*. This view is further strengthened by the fact that in response to salinity, the concentration of ABA has been shown to be significantly increased and that of IAA to be maintained in the salt-resistant maize but not in the salt-sensitive maize [82]. Moreover, the expression of the ABA responsive genes and the genes involved in ABA signaling are upregulated in the halophytes *M*. *crystallinum* in response to salt application [34].

An analysis of the transcriptome data revealed that both G protein and phosphoinositide signaling, two important membrane-associated signaling pathways, were operative in *S*. *maritima*, and the significant change in the expression of the individual components in response to the salt application (Fig 10) suggests that these play an important role in salt tolerance in this plant. A knock-out mutation of *GCR1* (G protein coupled receptor) has been reported to improve salt tolerance in *Arabidopsis* [83], which is in contrast to the significant upregulation of the *GCR1* observed in the present study (Fig 10), suggesting that G protein signaling with regard to salt tolerance may be different in halophytes and glycophytes. However, it has been reported that the expression of the G*α* and G*β* genes of the heterotrimeric G proteins is increased by NaCl treatment in rice [84–85], and their overexpression confers tolerance to salt in transgenic tobacco [86], which is in contrast to the report of a mutation of *GCR1* and *GPA1* (G protein *α* subunit) improving salt tolerance in *Arabidopsis* [83]. A mutation in G*α* protein has also been reported to reduce leaf senescence and chlorophyll degradation in maize under salt stress [87]. Mutation and transgenic studies have thus shown contrasting results with regard to the role of G protein signaling in salt tolerance in plants. However, with regard to small G protein signaling, salt tolerance seems to be improved by its inhibition, as demonstrated by the significant salt-induced upregulation of the GTPase activating protein (*GAP*) in the present study (Fig 10), which is in agreement with the findings of a report showing enhanced salt tolerance in *GCR1* and *GPA* mutants because an increase in GAP would result in the accumulation of its inactive GDP-bound form. However, information on the response of small G protein signaling to salt application is scant.

Signaling mediated by both phospholipase C, including PI (phosphoinositide)-phospholipase (PI-PLC) and non-specific phospholipase (NPC), and phospholipase D (PLD) were operative in *S*. *maritima* as shown by the expression of these enzymes (Fig 10). The signaling product of the reaction catalyzed by phospholipase D and NPC is phosphatidic acid (PA) [88], and it seems to be generated in *S*. *maritima* mainly by NPC in the presence of salt as the expression of both *DGK*, which converts diacylgylcerol (DAG) to PA, and *PLD* were downregulated in response to the salt treatment (Fig 10). In contrast, the expression of *PLD* has been reported to be enhanced in *Arabidopsis* upon exposure to high salinity together with an increase in its activity and the tissue accumulation of PA [89]. Similarly, *PLD* expression is upregulated in tomato cell suspension cultures treated with salt, and *PLD* knock-out *Arabidopsis* mutants exhibit enhanced sensitivity to high salinity [90], indicating an important role of the enzyme in salt tolerance in plants, at least in glycophytes. Unlike the decrease in *PLD* expression, the increased expression of *NPC* observed in *S*. *maritima* in response to NaCl application is in agreement with its reported enhancement following salt treatment in *Arabidopsis* [91], and hence, it could be important for salt tolerance, although the downregulation of *DGK* in response to salt treatment in the present study (Fig 10) does not support this. Rather, it seems that in *S*. *maritima*, *PI-PLC* may be playing an important role in salt tolerance. This is not only evident from its significant upregulation in response to NaCl application but also from the significant upregulation of protein kinase C (*PKC*), which targets DAG. *PKC* has, however, not been reported to be present in any higher plant genomes sequenced so far [92]. This is the first report of the presence and expression of *PKC* in the plant kingdom.

## Conclusion

**Fig 13:**
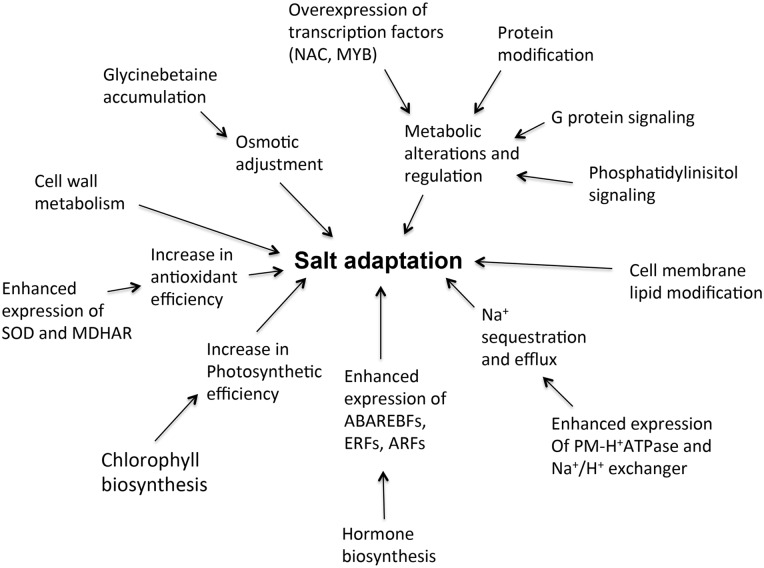
Schematic representation of the mechanisms possibly leading to salt-adaptation in plants based on sequencing and experimental data.

This study thus revealed that salt tolerance in *S*. *maritima* is the result of biochemical and molecular responses at multiple levels (Fig 13). It is difficult to say which are the first response and which are the most important among them, but it is likely that the response at each level contributes to salt tolerance in some way. It may be that one or more of these responses are the results of adaptations of *S*. *maritima* to high salinity rather than the cause of these adaptations. The increase in chlorophyll biosynthesis and photosynthetic efficiency might be among these. However, delignification and lignification of the cell wall and cell wall synthesis per se, as well as cell membrane lipid modifications, must be adaptive responses to high salinity. The importance of processes related to cell wall metabolism, which facilitate cell wall expansion through its remodeling [93], in salt tolerance is reflected by the fact that under salt stress the salt-tolerant variety of maize develops more extensible cell walls than salt-sensitive maize, which develops stiffer cell walls under salt stress compared with those of the control plants [94]. Similarly, the plasma membrane fatty acid unsaturation index increases upon the exposure of plants to salinity [95], and the unsaturation of fatty acids in the membrane lipids, in which fatty acid desaturase may play an important role (S11 File), significantly enhances the tolerance of the photosynthetic machinery in *Synechococcus* sp. to increased salinity [96]. Glycinebetaine accumulation and increases in the antioxidant capacity are well-studied strategies for protection against salt and osmotic stresses in plants [16,60], and hence, these might be of importance for the salt tolerance in *S*. *maritima* discussed in the present study. In addition, because salt has ionic components, particularly Na^+^, its sequestration in the cell and/or exclusion have been suggested to be of prime importance for salt tolerance [12], which in the present study is reflected as an enhancement in the expression of *PM-H*^*+*^*ATPase* and the Na^+^/H^+^ antiporter in response to salt application. The salt-induced upregulation of genes encoding the protein degradation and modification machinery, however, show that salt tolerance might be even far more complex, requiring the maintenance of specific proteins at the required levels and in their required form to drive necessary biochemical reactions. The complexity of the processes leading to salt tolerance is also reflected by the enhanced expression of transcription factors, such as *NAC* and *HD-Zip*, and by the enhanced expression of the various hormone responsive factors, which might regulate a wide range of chemical reactions and molecular events leading to salt tolerance. The initial response to salt application or the initial stage of the salt tolerance process, however, might be driven or regulated by G protein and phosphoinositide signaling, as is indicated by the upregulation of the genes encoding proteins contributing to these signaling events.

## Materials and Methods

### Test plant and NaCl application

Seeds of *S*. *maritima* L. were collected during the seed-setting season from an adult plant growing along the seashore of the mangrove coastal belt, Bhadrak in Odisha (21.13°N, 86.76°E), India. *S*. *maritima* species is not an endangered or a protected species. No specific permissions were required for collecting *S*. *maritima* seeds from the study location, as it is not a protected area of land or sea and because the field studies did not involve endangered or protected species. The seeds were spread on autoclaved soil in plastic pots with holes at the bottom and watered every day alternatively with 1/10th Hoagland’s solution or Milli-Q water. The seedlings were grown in a growth chamber, which was maintained at 24 ± 3°C with 70–75% relative humidity and a 14 h light (200 μmol m^-2^ s^-1^)/10 h dark cycle. After 3–4 weeks the seedlings were approximately 2 cm in height. These were transferred to soil in plastic pots with holes at the bottom. The seedlings were allowed to acclimatize and grow for ~3 months under natural day/night cycles in a greenhouse, which was maintained at 24 ± 3°C and 70–75% relative humidity. The individual pots were watered every day alternatively with 1/10th Hoagland’s solution or Milli-Q water except on the penultimate day of NaCl application. For the NaCl application, initially 500 mL of 0.5% NaCl, which was prepared in 1/10th strength Hoagland’s solution, was poured into each pot early in the morning. The control pots received only 1/10th Hoagland’s solution. After 30 minutes, when all of 0.5% NaCl solution drained away, 100 mL of 2.0% NaCl prepared in 1/10th strength Hoagland’s solution was poured into the treated pots in 30-minute intervals to maintain the concentration of the NaCl treatment at 2.0%. An initial treatment of 0.5% NaCl was applied for a brief period to prevent the plants from experiencing a sudden salinity shock. After 9 h of exposure to 2.0% NaCl, the shoots of the test plants were excised and deep-frozen in liquid nitrogen. These were stored at -80°C until analysis.

### RNA extraction, cDNA synthesis, library preparation and sequencing

Total RNA was extracted from the preserved shoot samples of the control and 2.0% NaCl-treated (9 h) *S*. *maritima* using an Illumina HiSeq 2000 and a TruSeq Library kit following the protocol in the manual. The quality and quantity of the extracted RNA was analyzed using an Agilent 2100 Bioanalyzer (Agilent Technologies). The extracted RNA with an RNA integrity number (RIN) of 8.0 was used for mRNA purification. mRNA was purified using oligo-dT beads (TruSeq RNA Sample Preparation Kit, Illumina) taking 1 μg of intact total RNA. The purified mRNA was fragmented at 90°C in the presence of divalent cations. The fragments were reverse transcribed using random hexamers and Superscript II Reverse Transcriptase (Life Technologies). Second strand cDNA was synthesized on the first strand template using RNaseH and DNA polymerase I. The cDNAs so obtained were cleaned using Beckman Coulter Agencourt Ampure XP SPRI beads. The cDNA molecules were ligated with Illumina adapters after end-repair and the addition of an ‘A’ base followed by SPRI cleanup. The prepared cDNA library was amplified using PCR for the enrichment of the adapter-ligated fragments. The individual libraries were quantified using a NanoDrop spectrophotometer (Thermo Scientific) and validated for quality with a Bioanalyzer (Agilent Technologies). These were then sequenced using the Illumina HiSeq 2000 platform. Paired-end FASTq files were subjected to standard quality control based on Phred scores >20 using the NGSQC Tool Kit to obtain high quality (HQ) filtered reads.

#### De novo transcriptome assembly and analysis

All the HQ filtered paired-end libraries were subjected to pooled de-novo transcriptome assembly using a De bruijn graph-based Trinity Assembler that was optimized for Illumina paired-end data and configurable for all computing capacities based on the criteria of a) a default K-mer value, b) less memory foot print, and c) reproducibility [97]. The assembled transcripts with sequence lengths longer than 200 bp were then clustered using the CD-HIT-EST tool to obtain the set of non-redundant transcripts. Clustered transcripts >200 bp were considered as secondary assemblies and were analyzed further for annotation and expression profiling. The secondary assembly transcripts sequences have been deposited at the NCBI as SRA file (SRA accession: SRP071624).

### Transcriptome annotation and quantification

Annotation for all the unique transcripts (>200 bp) was performed using a BLASTX homology search against the Plant Genome Database (PlantGDB, http://www.plantgdb.org/) and the NCBI non-redundant (nr) protein Database (http://blast.ncbi.nlm.nih.gov/Blast.cgi?PAGE=Proteins). BLAST hits with e-value scores ≤ 0.001 and query coverages above 50% were considered as annotated homologous proteins, and the AWK script was used for filtering the reciprocal best hits. The BLAST hits were processed, and the associated Gene Ontology (GO) terms describing the biological process, molecular function and cellular component categories were retrieved. GO annotations were incorporated following specific gene identifiers and accession numbers. The expression levels of the transcripts in the individual libraries (control and treated) were assessed by mapping the HQ filtered reads using Bowtie 2 [98]. Mapped reads were further normalized using the reads per kilo base per million (RPKM) method.

### Differential gene expression analysis and biological analysis of differentially expressed genes (DEGs)

The differential expression of the expressed transcripts was assessed using DESeq software [99] based on the R programming environment. DESeq takes into consideration total number of reads in a library vs. total number of reads mapped to a particular transcript of the library in both control and treated to compute *p*-value using Student t-test. Transcripts that showed changes 2-fold or greater or that showed an up- or down-regulation with a *p* value (derived via student t-tests) of ≤ 0.05 were considered to be significantly differentially expressed. The unsupervised hierarchical clustering of up- and down-regulated genes was performed using Cluster 3.0 software (http://bonsai.hgc.jp/~mdehoon/software/cluster/software.htm) by applying the Pearson Uncentered Algorithm with Average Linkage Rule. Furthermore, transcript clusters were visualized using Java Tree View software (http://jtreeview.sourceforge.net/) to identify the patterns of up- and down-regulated transcripts. Biological analyses of the DEGs were performed 1) based on GO annotations obtained from the EBI-GOA database (http://www.ebi.ac.uk/GOA), 2) by comparing the protein sequences to functional classifications against the Cluster of Orthologous Groups (COGs) database (http://www.ncbi.nlm.nih.gov/COG/), and 3) by using the MapMan tool (http://mapman.gabipd.org/web/guest/mapman). For the MapMan analysis, BIN level information with the gene identifier (TAIR ID) was obtained from their database. All the *S*. *maritima* expressed transcripts were annotated with *Arabidopsis* homologs (from the TAIR database) and imported into the MapMan tool along with their fold change for the visualization of various pathway BINs. The MapMan analysis was performed to identify the functionally enriched BINs along with the genes involved in them and their fold change in expression.

### Real-time PCR analysis

Real-time PCR (qPCR) assays were carried out to determine the changes in the expression of selected genes in response to the 2.0% NaCl treatment (9 h). Total RNA isolated from the preserved shoot samples of control and 2.0% NaCl-treated (9 h) *S*. *maritima* as described above was used to prepare cDNA using the SuperScript^®^III First-Strand Synthesis kit from Invitrogen according to the manufacturer’s instructions. To make the cDNA, 1 μg of DNase-treated total RNA, 1 μL dNTP mix (10 mM each), and 1 μL oligo(dT)20 (50 μM) were added into 0.2 mL PCR tubes and incubated at 65°C for 5 min, followed by an incubation on ice for ~5 minutes. In the same tube, 4 μL of 5X First Strand Buffer, 1 μL 0.1 M DTT, 1 μL RNaseOUT (40 units/μL) and 1 μL (200 units) of SuperScript III Reverse Transcriptase were added in a final volume of 20 μL. Next, the contents of the PCR tube were incubated at 50°C for 1 h, followed by an incubation at 70°C for 15 min and finally at 4°C for 5 min. The prepared first strand cDNA was used as a template for qPCR using a QuantiTect SYBR Green PCR Kit according to the manufacturer’s instructions. Primers for the individual ESTs/genes (S13 Table) were designed using the online software Primer3web (http://bioinfo.ut.ee/primer3/). PCR amplification was carried out on LightCycler^®^ 480II (Roche) with the reaction cycles set as follows: 95°C for 5 min, followed by 40 cycles of 95°C for 30 s, 60°C for 30 s and 72°C for 30 s. PCR reactions for each selected gene in a biological sample (cDNA preparation) were performed in triplicate, and cDNA prepared from two biological samples were used for each gene. Each PCR set-up included a template- free well. The expression of the actin gene in each sample was used for normalization. The expression of a selected gene in the NaCl-treated plants relative to that in the control plant was calculated using the 2-ΔΔCT method [100]. Paired t-tests were performed to determine significant differences (*p* ≤ 0.05) in transcript abundance of selected genes between the control and NaCl-treated plants.

## Supporting Information

S1 File Quality distribution graph for control and treated samples for both paired-end reads R1 and R2 depicting maximum reads to lie above a Phred score of 20. (TIF)

S2 File Annotation of the secondary assembly transcripts based on a BLASTX search and their homology to the protein database of other species. (XLSX)

S3 File Top ten plant species showing maximum homology to the *S*. *maritima* genome based on a BLASTX search of the secondary assembly transcripts. (TIF)

S4 File Differential expression of genes in *S*. *maritima* in response to NaCl application. (XLSX)

S5 File Top 20 genes that were upregulated (A) and downregulated (B) in *S*. *maritima* in response to NaCl application. (TIF)

S6 File Heat map showing differential expression of the genes encoding transcription factors identified in the study under the control and NaCl treatment conditions. (PNG)

S7 File Heat map showing differential expression of the genes encoding proteins involved in the ubiquitin mediated degradation of proteins under the control and NaCl treatment conditions. (PNG)

S8 File Heat map showing differential expression of the genes encoding enzymes involved in osmolytes and hormone biosynthesis under the control and NaCl treatment conditions. (PNG)

S9 File Heat map showing differential expression of the genes encoding chaperons under the control and NaCl treatment conditions. (PNG)

S10 File Heat map showing differential expression of the genes encoding enzymes and proteins involved in post-transcriptional modification under the control and NaCl treatment conditions. (PNG)

S11 File Genes showing 2-fold or greater changes and/or showing significant differential expression at *p* ≤ 0.05 that were assigned to different BINs of the MapMan metabolic pathways. (XLSX)

S12 File EST sequences of the genes showing 2-fold or greater changes and/or showing significant differential expression at *p* ≤ 0.05 that were assigned to different BINs of the MapMan metabolic pathways. (XLSX)

S13 File Primer sequences used in the study. (XLSX)

## References

[1] Scharf K-D, Berberich T, Ebersberger I, Nover L. The plant heat stress transcription factor (Hsf) family: Structure, function and evolution. Biochim Biophys Acta 2012; 1819:104–119. 10.1016/j.bbagrm.2011.10.002 22033015

[2] Zhu JK. Salt and drought stress signal transduction in plants. Ann Rev Plant Biol 2002; 53:247–273. 10.1146/annurev.arplant.53.091401.143329 12221975 PMC3128348

[3] Munns R. Genes and salt tolerance: bringing them together. New Phytol 2005; 167:645–663. 10.1111/j.1469-8137.2005.01487.x 16101905

[4] Flowers TJ, Muscolo A. Introduction to the special issue: halophytes in a changing world. AoB Plants 2015; 7:plv020 10.1093/aobpla/plv020 2015. 25757984 PMC4422832

[5] Wyn-Jones RG. Slat tolerance In: Johnson CB, editor. Physiological processes limiting plant productivity. London:Butterworth 1981; pp. 271–292.

[6] Munns R, Tester M. Mechanisms of salinity tolerance. Ann Rev Plant Biol 2008; 59:651–681. 10.1146/annurev.arplant.59.032607.092911 18444910

[7] Sun W, Xu X, Zhu H, Liu A, Liu L, Li J, et al Comparative transcriptomic profiling of a salt-tolerant wild tomato species and a salt-sensitive tomato cultivar. Plant Cell Physiol 2010; 51:997–1006. 10.1093/pcp/pcq056 20410049

[8] Glenn EP, Brown JJ, Blumwald E. Salt tolerance and crop potential of halophytes. Crit Rev Plant Sci 1999; 18:227–255.

[9] Shaw BP, Rout NP, Barman BC, Choudhury SB, Rao KH. Distribution of macrophytic vegetation in relation to salinity in the Chilka lake, a lagoon along east-coast of India. Ind J Mar Sci 2000; 29:144–148.

[10] Flowers TJ, Troke PF, Yeo AR. The mechanism of salt tolerance in halophytes. Ann Rev Plant Physiol 1977; 28:89–121. 10.1146/annurev.pp.28.060177.000513

[11] Glenn E, Miyamoto M, Moore D, Brown JJ, Thompson TL, Brown P. Water requirements for cultivating *Salicornia bigelovii* Torr. with seawater on sand in a coastal desert environment. J Arid Environ 1997; 36:711–730. 10.1006/jare.1997.0253

[12] Greenway H, Munns R. Mechanisms of salt tolerance in nonhalophytes. Ann Rev Plant Physiol 1980; 31:149–190. 10.1146/annurev.pp.31.060180.001053

[13] Sahu BB, Shaw BP. Isolation, identification and expression analysis of salt-induced genes in *Suaeda maritima*, a natural halophyte, using PCR-based suppression subtractive hybridization. BMC Plant Biol 2009a; 9:69 10.1186/1471-2229-9-69 19497134 PMC2702304

[14] Yi X, Sun Y, Yang Q, Guo A, Chang L, Wang D, et al Quantitative proteomics of *Sesuvium portulacastrum* leaves revealed that ion transportation by V-ATPase and sugar accumulation in chloroplast played crucial roles in halophyte salt tolerance. J Proteomics 2014; 99:84–100. 10.1016/j.jprot.2014.01.017 24487036

[15] Shi HZ, Quintero FJ, Pardo JM, Zhu JK. The putative plasma membrane Na+/H+ antiporter SOS1 controls long-distance Na+ transport in plants. Plant Cell 2002; 14:465–477. 10.1105/tpc.010371 11884687 PMC152925

[16] Rout NP, Shaw BP. Salt tolerance in aquatic macrophytes: possible involvement of the antioxidative enzymes. Plant Sci 2001; 160:415–423. 10.1016/S0168-9452(00)00406-4 11166427

[17] Wong CE, Li Y, Labbe A, Guevara D, Nuin P, Whitty B, et al Transcriptional profiling implicates novel interactions between abiotic stress and hormonal responses in *Thellungiella*, a close relative of *Arabidopsis*. Plant Physiol 2006; 140:1437–1450. 10.1104/pp.105.070508 16500996 PMC1435811

[18] Koyama ML, Levesley A, Koebner RMD, Flowers TJ, Yeo AR. Quantitative trait loci for component physiological traits determining salt tolerance in rice. Plant Physiol 2001; 125:406–422. 10.1104/pp.125.1.406 11154348 PMC61021

[19] Tang X, Wang H, Shao C, Shao H. Global gene expression of *Kosteletzkya virginica* seedlings responding to salt stress. PLoS ONE 2015; 10:e0124421 10.1371/journal.pone.0124421 25901608 PMC4406580

[20] Serrano R. Salt tolerance in plants and microorganisms: toxicity targets and defense responses. Int Rev Cyt 1996; 165:1–52. 10.1016/S0074-7696(08)62219-6 8900956

[21] Liska AJ, Shevchenko A, Pick U, Katz A. Enhanced photosynthesis and redox energy production contribute to salinity tolerance in *Dunaliella* as revealed by homology-based proteomics. Plant Physiol 2004; 136:2806–2817. 10.1104/pp.104.039438 15333751 PMC523343

[22] Mazel A, Leshem Y, Tiwari BS, Levine A. Induction of salt and osmotic stress tolerance by overexpression of an intracellular vesicle trafficking protein AtRab7 (AtRabG3e). Plant Physiol 2004; 134:118–128. 10.1104/pp.103.025379 14657401 PMC316292

[23] Mishra NS, Tuteja R, Tuteja N. Signaling through MAP kinase networks in plants. Arch Biochem Biophys 2006; 452:55–68. 10.1016/j.abb.2006.05.001 16806044

[24] Swindell WR, Huebner M, Weber AP. Transcription profiling of Arabidopsis heat-shock proteins and transcription factors reveals extensive overlap between heat and non-heat stress response pathways. BMC Genomics 2007; 8:125 10.1186/1471-2164-8-125 17519032 PMC1887538

[25] Chourey K, Ramani S, Apte SK. Accumulation of LEA proteins in salt (NaCl) stressed young seedlings of rice (*Oryza sativa* L.) cultivar Bura Rata and their degradation during recovery from salinity stress. J Plant Physiol 2003; 160:1165–1174. 10.1078/0176-1617-00909 14610885

[26] Moons A, Prinsen E, Bauw G, Motagu MV. Antagonistic effects of abscisic acid and jasmonates on salt stress-inducible transcripts in rice roots. Plant Cell 1997; 9:2243–2259. 10.1105/tpc.9.12.2243 9437865 PMC157071

[27] Yang O, Popova OV, Süthoff U, Lüking I, Dietz K-J, Golldack D. The Arabidopsis basic leucine zipper transcription factor AtbZIP24 regulates complex transcriptional networks involved in abiotic stress resistance. Gene 2009; 436:45–55. 10.1016/j.gene.2009.02.010 19248824

[28] Tran L-SP, Nakashima K, Sakuma Y, Simpson SD, Fujita Y, Maruyama K, et al Isolation and functional analysis of *Arabidopsis* stress-inducible NAC transcription factors that bind to a drought-responsive cis-element in the early responsive to dehydration stress 1 promoter. Plant Cell 2004; 16:2481–2498. 10.1105/tpc.104.022699 15319476 PMC520947

[29] Yong H-Y, Zou Z, Kok E-P, Kwan B-H, Chow K, Nasu S, et al Comparative transcriptome analysis of leaves and roots in response to sudden increase in salinity in *Brassica napus* by RNA-seq. BioMed Res Intern 2014 10.1155/2014/467395 25177691 PMC4142189

[30] Flowers TJ, Galal HK, Bromham L. Evolution of halophytes: multiple origins of salt tolerance in land plants. Func Plant Biol 2010; 37:604–612. 10.1071/FP09269

[31] Yamamoto N, Takano T, Tanaka K, Ishige T, Terashima S, Endo C, et al Comprehensive analysis of transcriptome response to salinity stress in the halophytic turf grass *Sporobolus virginicus*. Front Plant Sci 2015; 6:241 10.3389/fpls.2015.00241 25954282 PMC4404951

[32] Garg R, Verma M, Agrawal S, Shankar R, Majee M, Jain M. Deep transcriptome sequencing of wild halophyte rice, *Porteresia coarctata*, provides novel insights into the salinity and submergence tolerance factors. DNA Res 2014; 21:69–84. 10.1093/dnares/dst042 24104396 PMC3925395

[33] Ma J, Zhang M, Xiao X, You J, Wang J, Wang T, et al Global transcriptome profiling of *Salicornia europaea* L. shoots under NaCl treatment. PLoS One 2013; 8:e65877 10.1371/journal.pone.0065877 23825526 PMC3692491

[34] Tsukagoshi H, Suzuki T, Nishikawa K, Agarie S, Ishiguro S, Higashiyama T. RNA-seq analysis of the response of the halophyte, *Mesembryanthemum crystallinum* (ice plant) to high salinity, PLoS One 2015; 10(2):e0118339 10.1371/journal.pone.0118339 25706745 PMC4338230

[35] Diray-Arce J, Clement M, Gul B, Khan MA, Nielsen BL. Transcriptome assembly, profiling and differential gene expression analysis of the halophyte *Suaeda fruticosa* provides insights into salt tolerance. BMC Genomics 2015; 16:353 10.1186/s12864-015-1553-x 25943316 PMC4422317

[36] Kong F, Li H, Sun P, Zhou Y, Mao Y. De Novo Assembly and characterization of the transcriptome of seagrass *Zostera marina* using Illumina paired-end sequencing. PLoS One 2014; 9(11):e112245 10.1371/journal.pone.0112245 25423588 PMC4244107

[37] Wang J, Li B, Meng Y, Ma X, Lai Y, Si E, et al Transcriptomic profiling of the salt-stress response in the halophyte *Halogeton glomeratus*. BMC Genomics 2015; 16:169 10.1186/s12864-015-1373-z 25880042 PMC4363069

[38] Bassett IJ, Crompton CW. The genus *Suaeda* (Chenopodiaceae) in Canada. Can J Bot 1978; 56:581–591.

[39] Wang SM, Zhang J, Flowers TJ. Low-affinity Na^+^ uptake in the halophyte *Suaeda maritima*. Plant Physiol 2007; 145:559–571. 10.1104/pp.107.104315 17766398 PMC2048717

[40] Huang J, Lu X, Zhang W, Huang R, Chen S, Zheng Y. Transcriptome sequencing and analysis of leaf tissue of *Avicennia marina* using the Illumina platform. PLoS One 2014; 9:e108785 10.1371/journal.pone.0108785 25265387 PMC4181315

[41] Liang C, Liu X, Yiu S-M, Lim BL. De novo assembly and characterization of *Camelina sativa* transcriptome by paired-end sequencing. BMC Genomics 2013; 14:146 10.1186/1471-2164-14-146 23496985 PMC3635884

[42] Sharma R, Mishra M, Gupta B, Parsania C, Singla-Pareek SL, Pareek A. De novo assembly and characterization of stress transcriptome in a salinity-tolerant variety CS52 of *Brassica juncea*. PLoS One 2015; 10:e0126783 10.1371/journal.pone.0126783 25970274 PMC4429966

[43] Hiz MC, Canher B, Niron H, Turet M. Transcriptome analysis of salt tolerant common bean (*Phaseolus vulgaris* L.) under saline conditions. PLoS One 2014; 9:e92598 10.1371/journal.pone.0092598 24651267 PMC3961409

[44] Xu P, Liu Z, Fan X, Gao J, Zhang X, Zhang X, et al De novo transcriptome sequencing and comparative analysis of differentially expressed genes in *Gossypium aridum* under salt stress. Gene 2013; 525:26–34. 10.1016/j.gene.2013.04.066 23651590

[45] Blasing OE, Gibon Y, Gunther M, Hohne M, Morcuende R, Osuna D, et al Sugars and circadian regulation make major contributions to the global regulation of diurnal gene expression in *Arabidopsis*. Plant Cell 2005; 17:3257–3281. 10.1105/tpc.105.035261 16299223 PMC1315368

[46] Walia H, Wilson C, Condamine P, Liu X, Ismail AM, Zeng L, et al Comparative transcriptional profiling of two contrasting rice genotypes under salinity stress during the vegetative growth stage. Plant Physiol 2005; 139:822–835. 10.1104/pp.105.065961 16183841 PMC1255998

[47] Zagorchev L, Kamenova P, Odjakova M. The role of plant cell wall proteins in response to salt stress. Scientific World J 2014 10.1155/2014/764089 24574917 PMC3916024

[48] Sandt VSTV, Guisez Y, Verbelen J-P, Vissenberg K. Analysis of a xyloglucan endotransglycosylase/hydrolase (XTH) from the lycopodiophyte *Selaginella kraussiana* suggests that XTH sequence characteristics and function are highly conserved during the evolution of vascular plants. J Exp Bot 2006; 57:2909–2922. 10.1093/jxb/erl064 16873447

[49] Nishikubo N, Takahashi J, Roos AA, Derba-Maceluch M, Piens K, Brumer H, et al Xyloglucan endo-transglycosylase-mediated xyloglucan rearrangements in developing wood of hybrid aspen. Plant Physiol 2011; 155:399–413. 10.1104/pp.110.166934 21057113 PMC3075792

[50] Cannon MC, Terneus K, Hall Q, Tan L, Wang Y, Wegenhart BL, et al Self-assembly of the plant cell wall requires an extensin scaffold. Proc Nat Acad Sci USA 2008; 105:2226–2231. 10.1073/pnas.0711980105 18256186 PMC2538902

[51] Gharat SA, Shaw BP. NaCl induced changes in the ionic and osmotic components in rice cultivars vis-a-vis that in a natural halophyte. Oryza 2015; 52:46–53.

[52] Miedes E, Vanholme R, Boerjan W, Molina A. The role of the secondary cell wall in plant resistance to pathogens. Front Plant Sci 2014; 5:358 10.3389/fpls.2014.00358 25161657 PMC4122179

[53] Madhavi V, Lele SS. Laccase: properties and applications. BioResources 2009; 4:1694–1717.

[54] Lam KC, Ibrahim RK, Behdad B, Dayanandan S. Structure, function, and evolution of plant O-methyltransferases. Genome 2007; 50:1001–1013. 10.1139/g07-077 18059546

[55] Pitzschke A, Datta S, Persak H. Salt stress in *Arabidopsis*: lipid transfer protein AZI1 and its control by mitogen-activated protein kinase MPK3. Mol Plant 2014; 7:722–738. 10.1093/mp/sst157 24214892 PMC3973493

[56] Zhang J, Liu H, Sun J, Li B, Zhu Q, Chen S, et al *Arabidopsis* fatty acid desaturase FAD2 is required for salt tolerance during seed germination and early seedling growth. PLoS One 2012; 7:e30355 10.1371/journal.pone.0030355 22279586 PMC3261201

[57] Liu D, Wang L, Zhai H, Song X, He S, Liu Q. A Novel a/b-hydrolase gene IbMas enhances salt tolerance in transgenic sweet potato. PLoS One 2014; 10.1371/journal.pone.0115128 25501819 PMC4264881

[58] Rontein D, David Rhodes D, Hanson AD. Evidence from engineering that decarboxylation of free serine is the major source of ethanolamine moieties in plants. Plant Cell Physiol 2003; 44:1185–1191. 10.1093/pcp/pcg144 14634155

[59] Moghaieb REA, Saneoka H, Fujita K. Effect of salinity on osmotic adjustment, glycinebetaine accumulation and the betaine aldehyde dehydrogenase gene expression in two halophytic plants, *Salicornia europaea* and *Suaeda maritima*. Plant Sci 2004; 166:1345–1349. 10.1016/j.plantsci.2004.01.016

[60] Gharat SA, Shaw BP. Novel and conserved miRNAs in the halophyte *Suaeda maritima* identified by deep sequencing and computational predictions using the ESTs of two mangrove plants. BMC Plant Biol 2015; 15:301 10.1186/s12870-015-0682-3 26714456 PMC4696257

[61] Stone SL. The role of ubiquitin and the 26S proteasome in plant abiotic stress signaling. Front Plant Sci 2014; 5:135 10.3389/fpls.2014.00135 24795732 PMC3997020

[62] Brini F, Hanin M, Lumbreras V, Irar S, Pages M, Masmoudi K. Functional characterization of DHN-5, a dehydrin showing a differential phosphorylation pattern in two Tunisian durum wheat (*Triticum durum* Desf.) varieties with marked differences in salt and drought tolerance. Plant Sci 2007; 172:20–28. 10.1016/j.plantsci.2006.07.011

[63] Tanou G, Job C, Rajjou L, Arc E, Belghazi M, Diamantidis G, et al Proteomics reveals the overlapping roles of hydrogen peroxide and nitric oxide in the acclimation of citrus plants to salinity. Plant J 2009; 60:795–804. 10.1111/j.1365-313X.2009.04000.x 19682288

[64] Shaw BP, Sahu SK, Mishra RK. Heavy metal induced oxidative damage in terrestrial plants In: Prasad MNV, editor. Heavy metal stress in plants. Heidelberg: Springer-Verlag; 2004 pp. 84–126. 10.1007/978-3-662-07743-6_4

[65] Puranik S, Sahu PP, Srivastava PS, Prasad M. NAC proteins: regulation and role in stress tolerance. Trends Plant Sci 2012; 6:369–381. 10.1016/j.tplants.2012.02.004 22445067

[66] Pascual MB, Cánovas FM, Ávila C. The NAC transcription factor family in maritime pine (*Pinus Pinaster*): molecular regulation of two genes involved in stress responses. BMC Plant Biol 2015; 15:254 10.1186/s12870-015-0640-0 26500018 PMC4619436

[67] Wang N, Zheng Y, Xin H, Fang L, Li S. Comprehensive analysis of NAC domain transcription factor gene family in *Vitis vinifera*. Plant Cell Rep 2013; 32:61–75. 10.1007/s00299-012-1340-y 22983198

[68] Nakashima K, Tran LS, Van Nguyen D, Fujita M, Maruyama K, Todaka D, et al Functional analysis of a NAC-type transcription factor OsNAC6 involved in abiotic and biotic stress-responsive gene expression in rice. Plant J 2007; 51:617–630. 10.1111/j.1365-313X.2007.03168.x 17587305

[69] Xiong H, Li J, Liu P, Duan J, Zhao Y, Guo X, et al Overexpression of osmyb48-1, a novel myb-related transcription factor, enhances drought and salinity tolerance in rice. PLoS One 2014; 9(3):e92913 10.1371/journal.pone.0092913 24667379 PMC3965499

[70] Katiyar A, Smita S, Lenka SK, Rajwanshi R, Chinnusamy V, Bansal KC. Genome-wide classification and expression analysis of MYB transcription factor families in rice and *Arabidopsis*. BMC Genomics 2012; 13:544 10.1186/1471-2164-13-544 23050870 PMC3542171

[71] He Y, Li W, Lv J, Jia J, Wang M, Xia G. Ectopic expression of a wheat MYB transcription factor gene, TaMYB73, improves salinity stress tolerance in *Arabidopsis thaliana*. J Exp Bot 2012; 63:1511–1522. 10.1093/jxb/err389 22140235

[72] Ganesan G, Sankararamasubramanaian HM, Harikrishnan M, Gapudi A, Parida A. A MYB transcription factor from the grey mangrove is induced by stress and confers NaCl tolerance in tobacco. J Exp Bot 2012; 63:4549–4561. 10.1093/jxb/ers135 22904269

[73] Olsson ASB, Engstrom P, Soderman E. The homeobox genes ATHB12 and ATHB7 encode potential regulators of growth in response to water deficit in *Arabidopsis*. Plant Mol Biol 2004; 55:663–677. 10.1007/s11103-004-1581-4 15604708

[74] Tan QKG, Irish VF. The Arabidopsis zinc finger-homeodomain genes encode proteins with unique biochemical properties that are coordinately expressed during floral development. Plant Physiol 2006; 140:1095–1108. 10.1104/pp.105.070565 16428600 PMC1400567

[75] Hur Y-S, Um J-H, Kim S, Kim K, Park H-J, Lim J-S, et al *Arabidopsis thaliana* homeobox 12 (ATHB12), a homeodomain-leucine zipper protein, regulates leaf growth by promoting cell expansion and endoreduplication, New Phytol 2015; 205:316–328. 10.1111/nph.12998 25187356

[76] Shin D, Koo YD, Lee J, Lee H-J, Baek D, Lee S, et al Athb-12, a homeobox-leucine zipper domain protein from *Arabidopsis thaliana*, increases salt tolerance in yeast by regulating sodium exclusion. Biochem Biophys Res Commun 2004; 323:534–540. 10.1016/j.bbrc.2004.08.127 15369784

[77] Söderman E, Hjellström M, Fahleson J, Engström P. The HD-Zip gene ATHB6 in *Arabidopsis* is expressed in developing leaves, roots and carpels and up-regulated by water deficit conditions. Plant Mol Biol 1999; 40:1073–1083. 10527431 10.1023/a:1006267013170

[78] Golldack D, Lüking I, Yang O. Plant tolerance to drought and salinity: stress regulating transcription factors and their functional significance in the cellular transcriptional network. Plant Cell Rep 2011; 30:1383–1391. 10.1007/s00299-011-1068-0 21476089

[79] Li SJ, Fu QT, Chen LG, Huang WD, Yu DQ. *Arabidopsis thaliana* WRKY25, WRKY26, and WRKY33 coordinate induction of plant thermotolerance. Planta 2011; 233:1237–1252. 10.1007/s00425-011-1375-2 21336597

[80] Panda BB, Badoghar AK, Sekhar S, Kariali E, Mohapatra PK, Shaw BP. Biochemical and molecular characterisation of salt-induced poor grain filling in a rice cultivar. Func Plant Biol 2015 10.1071/FP15229 32480459

[81] Shen X, Wang Z, Song X, Xu J, Jiang C, Zhao Y, et al Transcriptomic profiling revealed an important role of cell wall remodeling and ethylene signaling pathway during salt acclimation in *Arabidopsis*. Plant Mol Biol 2014; 86:303–317. 10.1007/s11103-014-0230-9 25092201

[82] Zörb C, Geilfus C-M, Mühling KH, Ludwig-Müller J. The influence of salt stress on ABA and auxin concentrations in two maize cultivars differing in salt resistance. J Plant Physiol 2013; 170:220–224. 10.1016/j.jplph.2012.09.012 23181973

[83] Chakraborty N, Singh N, Kaur K, Raghuram N. G-protein signaling components GCR1 and GPA1 mediate responses to multiple abiotic stresses in *Arabidopsis*. Front Plant Sci 2015; 6:1000 10.3389/fpls.2015.01000 26635828 PMC4649046

[84] Yadav DK, Islam SMS, Tuteja N. Rice heterotrimeric G-protein Gamma subunits (RGG1 and RGG2) are differentially regulated under abiotic stress. Plant Signal Behav 2012; 7:733–740. 10.4161/psb.20356 22751322 PMC3583952

[85] Yadav D, Shukla D, Tuteja N. Rice heterotrimeric G-protein alpha subunit (RGA1): in silico analysis of the gene and promoter and its upregulation under abiotic stress. Plant Physiol Biochem 2013; 63:262–271. 10.1016/j.plaphy.2012.11.031 23313793

[86] Misra S, Wu Y, Venkataraman G, Sopory S, Tuteja N. Heterotrimeric G-protein complex and G-protein-coupled receptor from a legume (*Pisum sativum*): role in salinity and heat stress and cross-talk with phospholipase C. Plant J 2007; 51:656–669. 10.1111/j.1365-313X.2007.03169.x 17587233

[87] Urano D, Colaneri A, Jones AM. Gα modulates salt-induced cellular senescence and cell division in rice and maize. J Exp Bot 2014; 10.1093/jxb/eru372 25227951 PMC4246186

[88] Testerink C, Munnik T. Phosphatidic acid: a multifunctional stress signaling lipid in plants. Trends Plant Sci 2005; 10:368–375. 10.1016/j.tplants.2005.06.002 16023886

[89] Katagiri T, Takahashi S, Shinozaki K. Involvement of a novel *Arabidopsis* phospholipase D, AtPLDd, in dehydration-inducible accumulation of phosphatidic acid in stress signaling, Plant J 2001; 26:595–605. 11489173 10.1046/j.1365-313x.2001.01060.x

[90] Bargmann BOR, Laxalt AM, Riet BT, Schooten BV, Merquiol E, Testerink C, et al Multiple PLDs required for high salinity and water deficit tolerance in plants. Plant Cell Physiol 2009; 50:78–89. 10.1093/pcp/pcn173 19017627 PMC2638713

[91] Peters C, Kim S-C, Devaiah S, Li M, Wang X. Non-specific phospholipase C5 and diacylglycerol promote lateral root development under mild salt stress in *Arabidopsis*. Plant Cell Environ 2014; 37:2002–2013. 10.1111/pce.12334 24689655

[92] Munnik T, Testerink C. Plant phospholipid signaling: “in a nutshell”. J Lipid Res 2009; 50:S260–S265. 10.1194/jlr.R800098-JLR200 19098305 PMC2674723

[93] Tenhaken R. Cell wall remodeling under abiotic stress. Front Plant Sci 2015; 10.3389/fpls.2014.00771 25709610 PMC4285730

[94] Zörb C, Mühling KH, Kutschera U, Geilfus C-M. Salinity stiffens the epidermal cell walls of salt-stressed maize leaves: is the epidermis growth-restricting? PLoS One 2015; 10(3):e0118406 10.1371/journal.pone.0118406 25760715 PMC4356557

[95] López-Pérez L, Martínez-Ballesta MdC, Maurel C, Carvajal M. Changes in plasma membrane lipids, aquaporins and proton pump of broccoli roots, as an adaptation mechanism to salinity. Phytochem 2009; 70:492–500. 10.1016/j.phytochem.2009.01.014 19264331

[96] Allakhverdiev SI, Kinoshita M, Inaba M, Suzuki I, Murata N. Unsaturated fatty acids in membrane lipids protect the photosynthetic machinery against salt-induced damage in *Synechococcus*. Plant Physiol 2001; 125:1842–1853. 10.1104/pp.125.4.1842 11299364 PMC88840

[97] Bankar KG, Todur VN, Shukla RN, Vasudevan M. Ameliorated de novo transcriptome assembly using Illumina paired end sequence data with Trinity Assembler. Genomics Data 2015; 5:352–359. 10.1016/j.gdata.2015.07.012 26484285 PMC4583709

[98] Langmead B, Salzberg SL. Fast gapped-read alignment with Bowtie 2. Nat Methods 2012; 9:357–359. 10.1038/nmeth.1923 22388286 PMC3322381

[99] Anders S, Huber W. Differential expression analysis for sequence count data. Genome Biol 2010; 11:R106 10.1186/gb-2010-11-10-r106 20979621 PMC3218662

[100] Livak KJ, Schmittgen TD. Analysis of relative gene expression data using real-time quantitative PCR and the 2(-Delta Delta C(T)) Method. Methods 2001; 25:402–408. 10.1006/meth.2001.1262 11846609

