# Supplementary figures and images for: Transcriptome Analysis of the Response to NaCl in Suaeda maritima Provides an Insight into Salt Tolerance Mechanisms in Halophytes
Source: PLoS One. 2016 Sep 28;11(9):e0163485. doi: 10.1371/journal.pone.0163485 (PMC5040429; doi:10.1371/journal.pone.0163485)

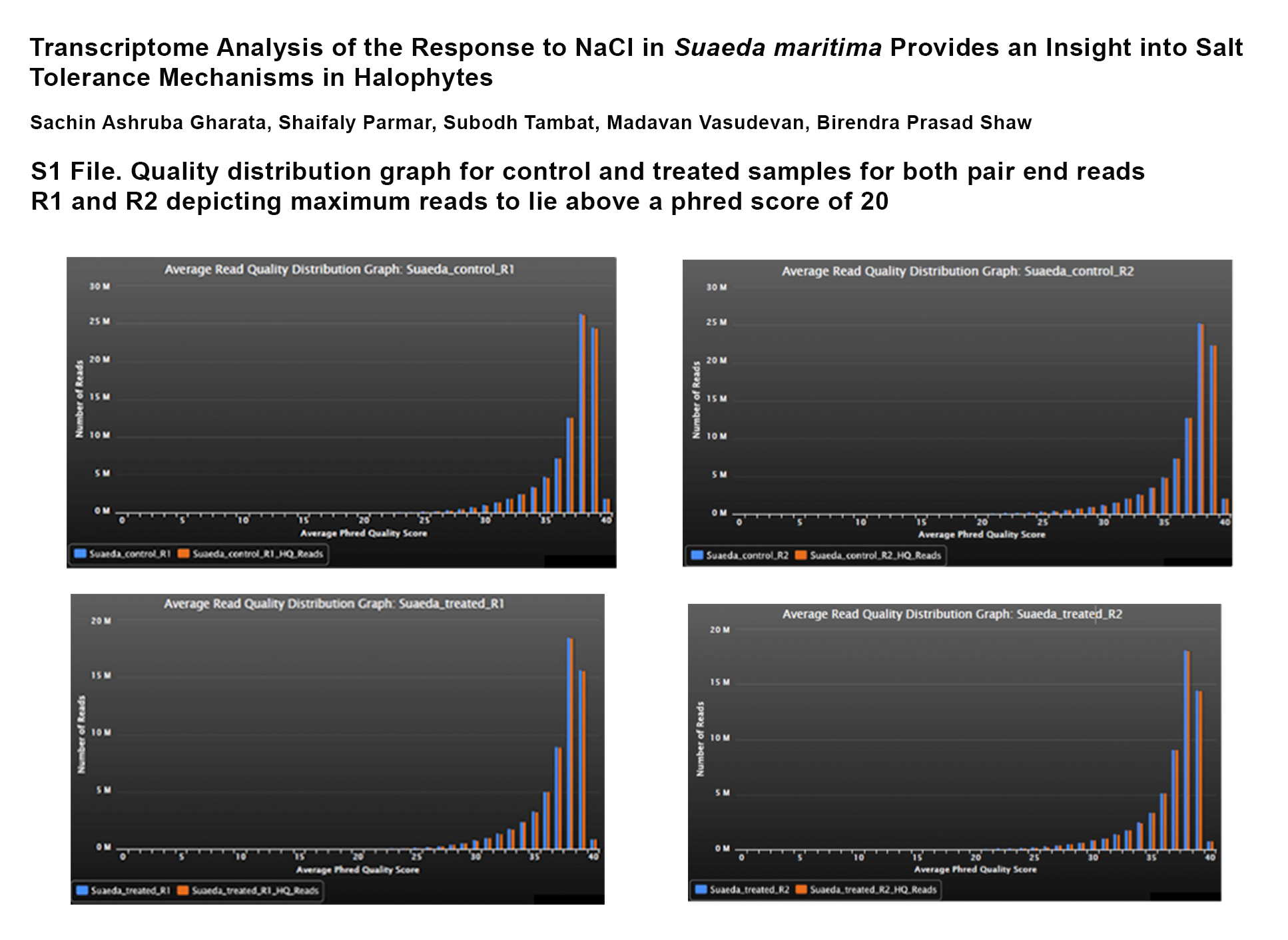

Supplement: S1 File — (TIF) [file pone.0163485.s001.tif]

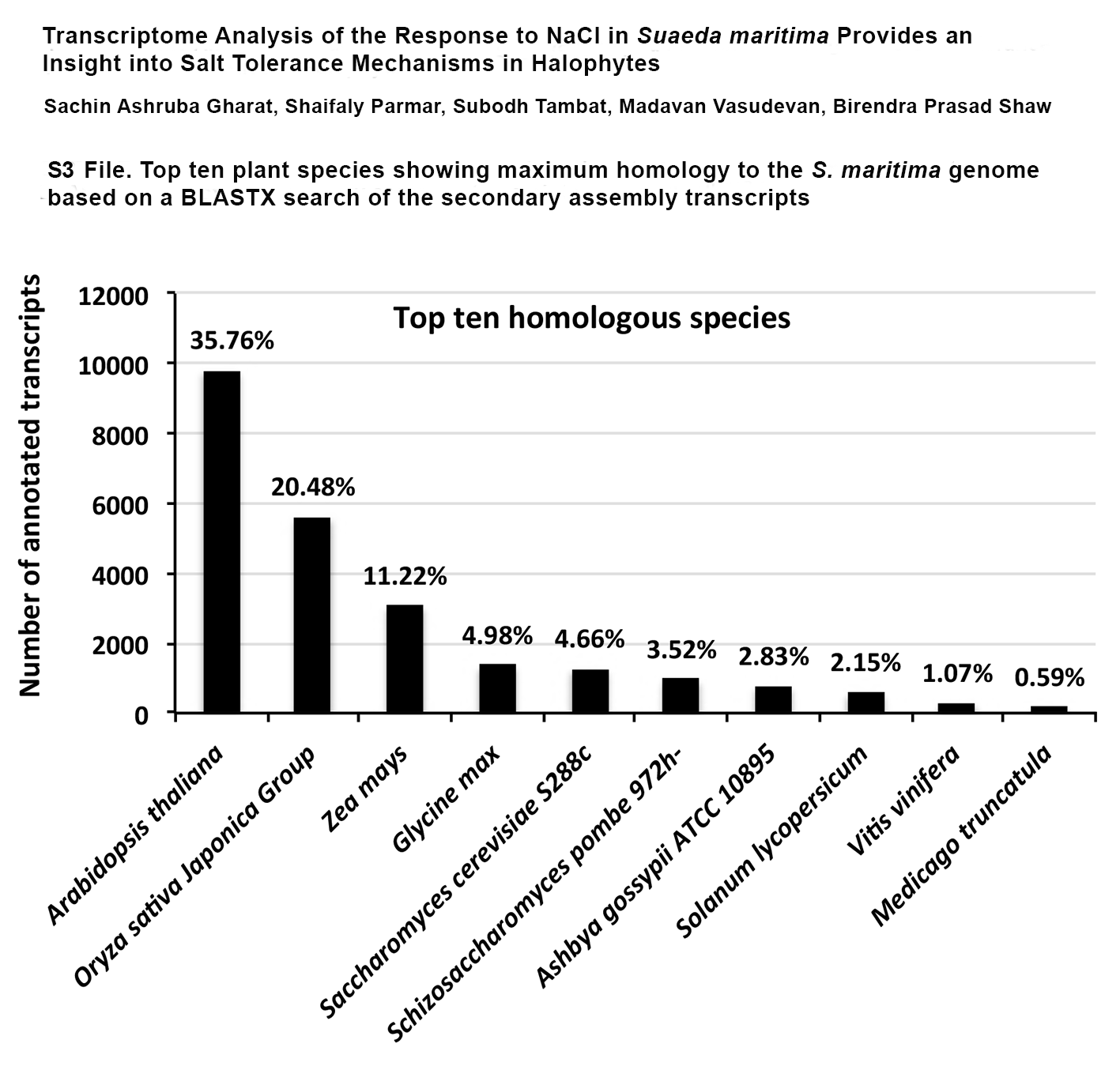

Supplement: S3 File — (TIF) [file pone.0163485.s003.tif]

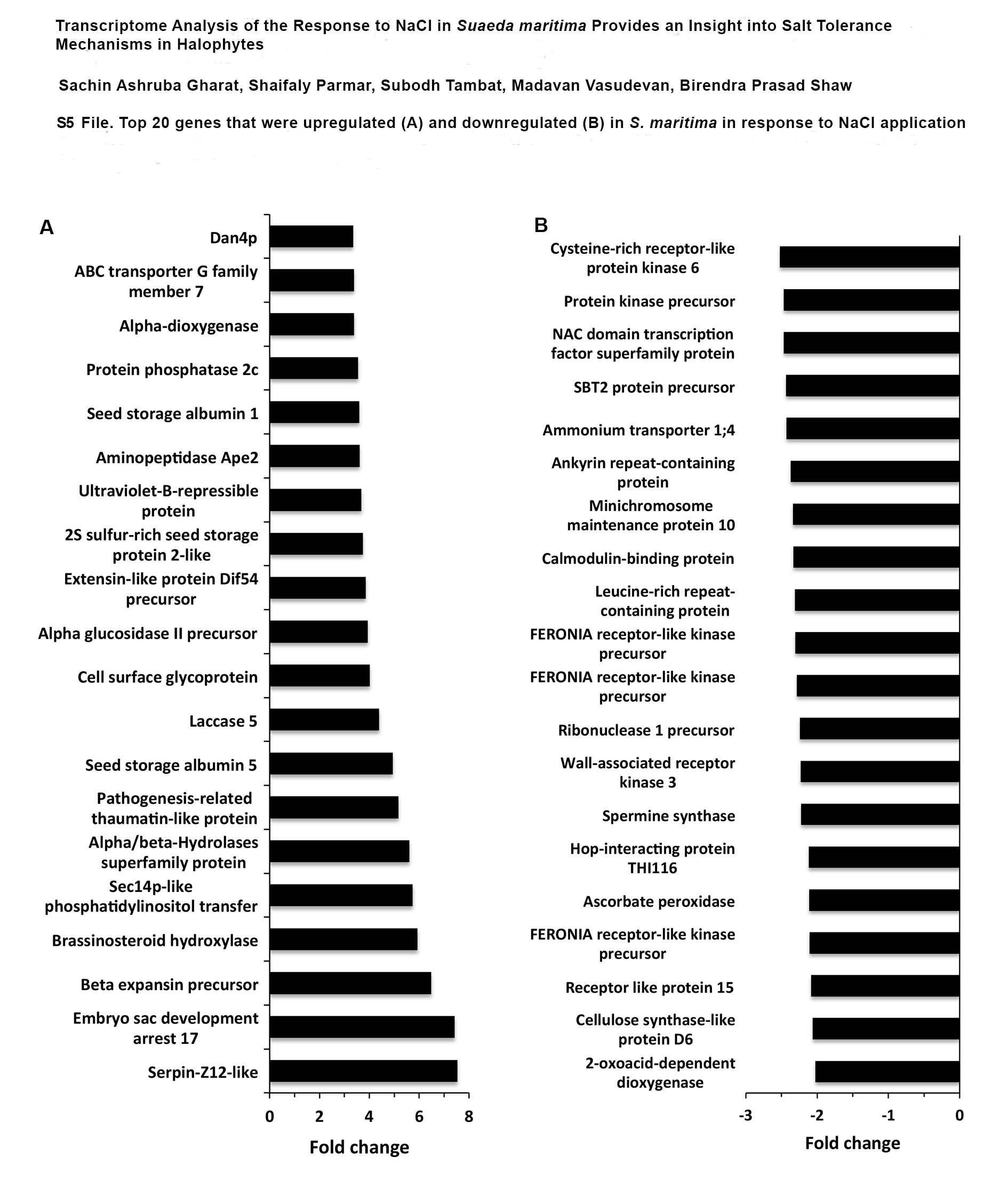

Supplement: S5 File — (TIF) [file pone.0163485.s005.tif]

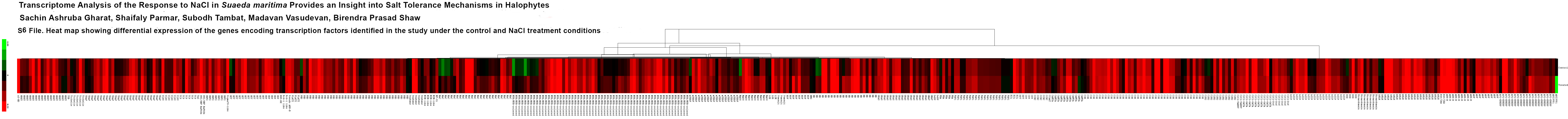

Supplement: S6 File — (PNG) [file pone.0163485.s006.png]

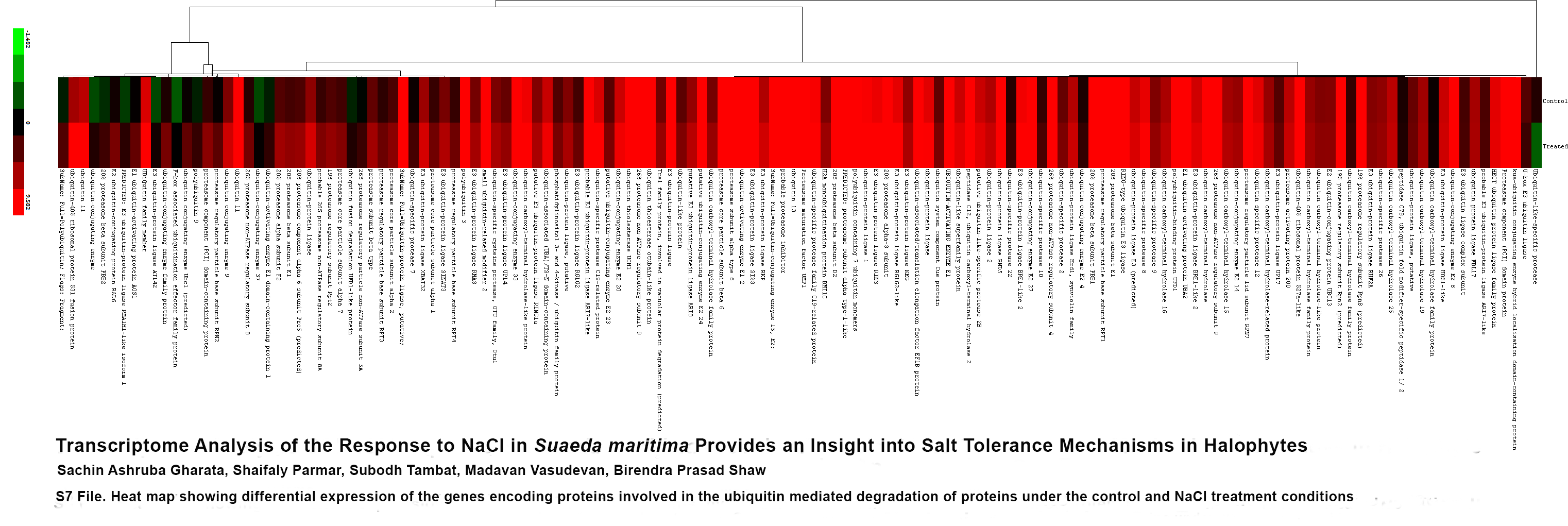

Supplement: S7 File — (PNG) [file pone.0163485.s007.png]

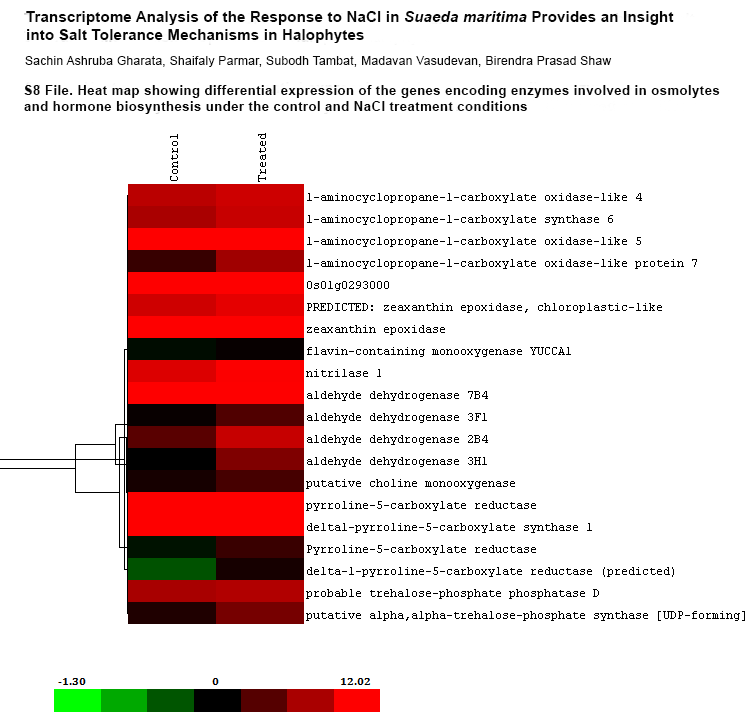

Supplement: S8 File — (PNG) [file pone.0163485.s008.png]

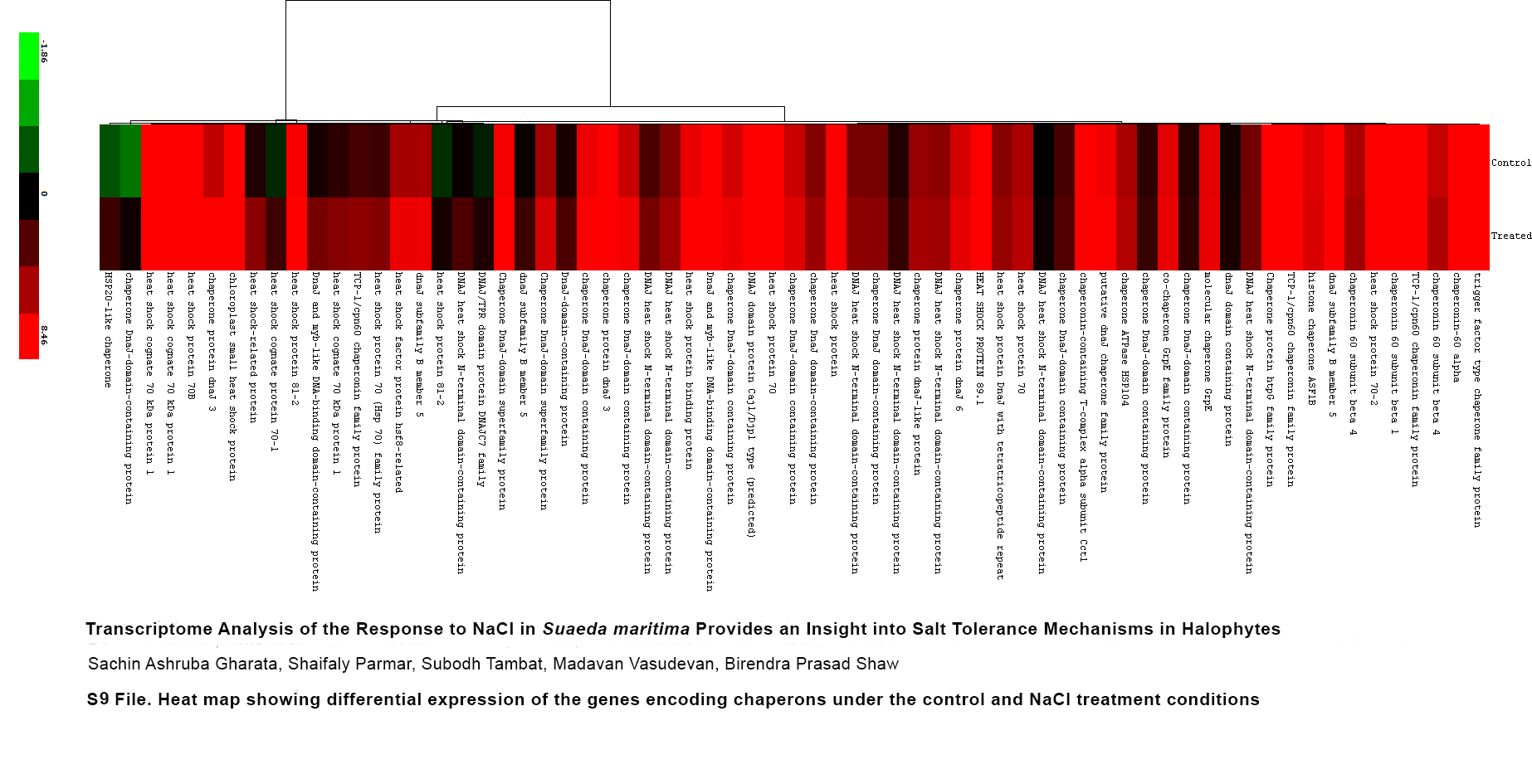

Supplement: S9 File — (PNG) [file pone.0163485.s009.png]

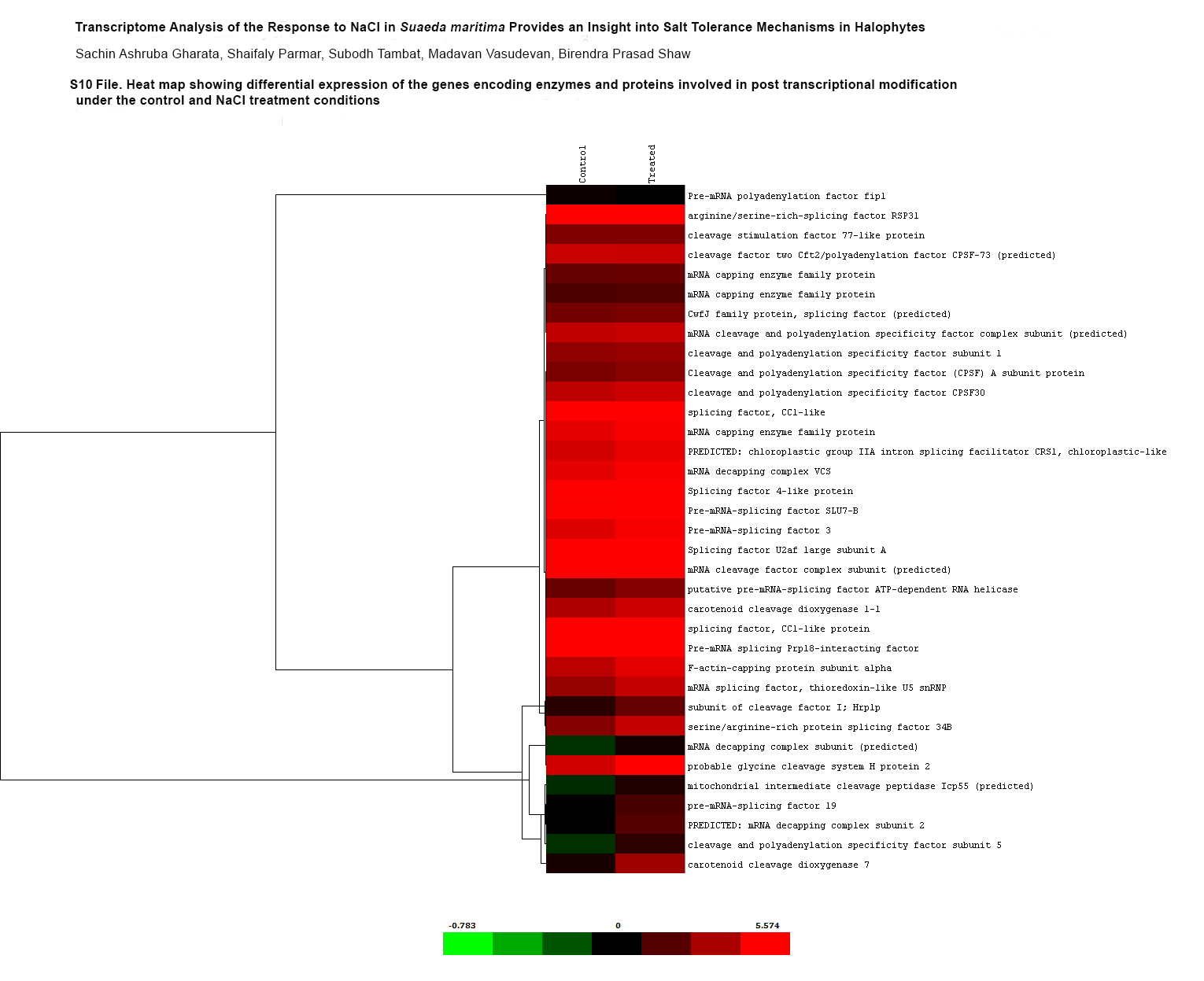

Supplement: S10 File — (PNG) [file pone.0163485.s010.png]
